# Nuclear factor kappa B-dependent persistence of *Salmonella* Typhi and Paratyphi in human macrophages

**DOI:** 10.1128/mbio.00454-24

**Published:** 2024-03-18

**Authors:** Taylor A. Stepien, Larissa A. Singletary, Fermin E. Guerra, Joyce E. Karlinsey, Stephen J. Libby, Sarah L. Jaslow, Margaret R. Gaggioli, Kyle D. Gibbs, Dennis C. Ko, Michael A. Brehm, Dale L. Greiner, Leonard D. Shultz, Ferric C. Fang

**Affiliations:** 1 Department of Global Health, University of Washington, Seattle, Washington, USA; 2 Department of Microbiology, University of Washington, Seattle, Washington, USA; 3 Department of Laboratory Medicine and Pathology, University of Washington, Seattle, Washington, USA; 4 Department of Molecular Genetics and Microbiology, Duke University, Durham, North Carolina, USA; 5 Department of Molecular Medicine, University of Massachusetts Medical School, Worcester, Massachusetts, USA; 6 The Jackson Laboratory, Bar Harbor, Maine, USA; University of Utah, Salt Lake City, Utah, USA

**Keywords:** *Salmonella*, typhoid, macrophages, apoptosis, human, pathogenesis

## Abstract

*Salmonella* serovars Typhi and Paratyphi cause a prolonged illness known as enteric fever, whereas other serovars cause acute gastroenteritis. Mechanisms responsible for the divergent clinical manifestations of nontyphoidal and enteric fever *Salmonella* infections have remained elusive. Here, we show that *S*. Typhi and *S*. Paratyphi A can persist within human macrophages, whereas *S*. Typhimurium rapidly induces apoptotic macrophage cell death that is dependent on *Salmonella* pathogenicity island 2 (SPI2). *S*. Typhi and *S*. Paratyphi A lack 12 specific SPI2 effectors with pro-apoptotic functions, including nine that target nuclear factor κB (NF-κB). Pharmacologic inhibition of NF-κB or heterologous expression of the SPI2 effectors GogA or GtgA restores apoptosis of *S*. Typhi-infected macrophages. In addition, the absence of the SPI2 effector SarA results in deficient signal transducer and activator of transcription 1 (STAT1) activation and interleukin 12 production, leading to impaired T_H_1 responses in macrophages and humanized mice. The absence of specific nontyphoidal SPI2 effectors may allow *S*. Typhi and *S*. Paratyphi A to cause chronic infections.

**IMPORTANCE:**

Salmonella enterica is a common cause of gastrointestinal infections worldwide. The serovars *Salmonella* Typhi and *Salmonella* Paratyphi A cause a distinctive systemic illness called enteric fever, whose pathogenesis is incompletely understood. Here, we show that enteric fever *Salmonella* serovars lack 12 specific virulence factors possessed by nontyphoidal *Salmonella* serovars, which allow the enteric fever serovars to persist within human macrophages. We propose that this fundamental difference in the interaction of *Salmonella* with human macrophages is responsible for the chronicity of typhoid and paratyphoid fever, suggesting that targeting the nuclear factor κB (NF-κB) complex responsible for macrophage survival could facilitate the clearance of persistent bacterial infections.

## INTRODUCTION

Enteric fever caused by typhoidal *Salmonella enterica* serovars Typhi and Paratyphi A is a life-threatening systemic illness afflicting up to 20 million persons each year, primarily in low- and middle-income countries with inadequate sanitation (1). As *S*. Typhi and *S*. Paratyphi A are host restricted for humans, most studies of systemic *Salmonella* infection have relied on murine models using nontyphoidal *S*. Typhimurium, which have identified conserved features of *Salmonella* virulence (2). However, *S*. Typhi and *S*. Paratyphi A differ from nontyphoidal *Salmonella* (NTS) with regard to both gene loss and acquisition (3–5), and the mechanistic basis for the different clinical syndromes caused by *Salmonella* serovars has remained elusive. Although a toxin produced by *S*. Typhi, *S*. Paratyphi A, and some nontyphoidal *Salmonella* serovars has been suggested to play an important pathogenic role in enteric fever (6), the typhoid toxin is not required for *S*. Typhi virulence in humanized mice (7) nor in a human challenge model (8). All *Salmonella enterica* serovars are thought to rely on type three secretion systems (T3SS) encoded by *Salmonella* pathogenicity islands 1 and 2 (SPI1 and SPI2) for host cell invasion and intracellular survival (9–11). Effector proteins secreted by SPI1 and SPI2 have been shown to remodel host cellular functions, subvert the immune response, establish the *Salmonella*-containing vacuole, and promote bacterial replication (12).

Both NTS and enteric fever *Salmonella* serovars are internalized by phagocytes, but a hallmark of enteric fever is intracellular bacterial survival with systemic dissemination of infected cells to distant sites (13, 14) and the persistence of infection (15). In contrast, *S*. Typhimurium and other NTS are largely confined to the intestine, where they elicit a brief and self-limited inflammatory diarrhea. *S*. Typhimurium induces apoptosis of infected macrophages, which is dependent on type 3 secretion and promotes bacterial survival (16, 17). Epidemiological evidence suggests that T_H_1 cellular immune responses and interferon gamma (IFNγ) signaling do not play a significant role in the incidence nor the severity of enteric fever (18, 19), despite their critical importance for the clearance of NTS infections (20, 21), suggesting a fundamental difference in the interaction of NTS and enteric fever *Salmonella* serovars with the human immune system. This study compares the interaction between *S*. Typhi and *S*. Typhimurium with human macrophages to gain insights into their contrasting pathogenic strategies.

## RESULTS

### Differential cytotoxicity of *S*. Typhi and *S*. Typhimurium for human macrophages

**Fig 1:**
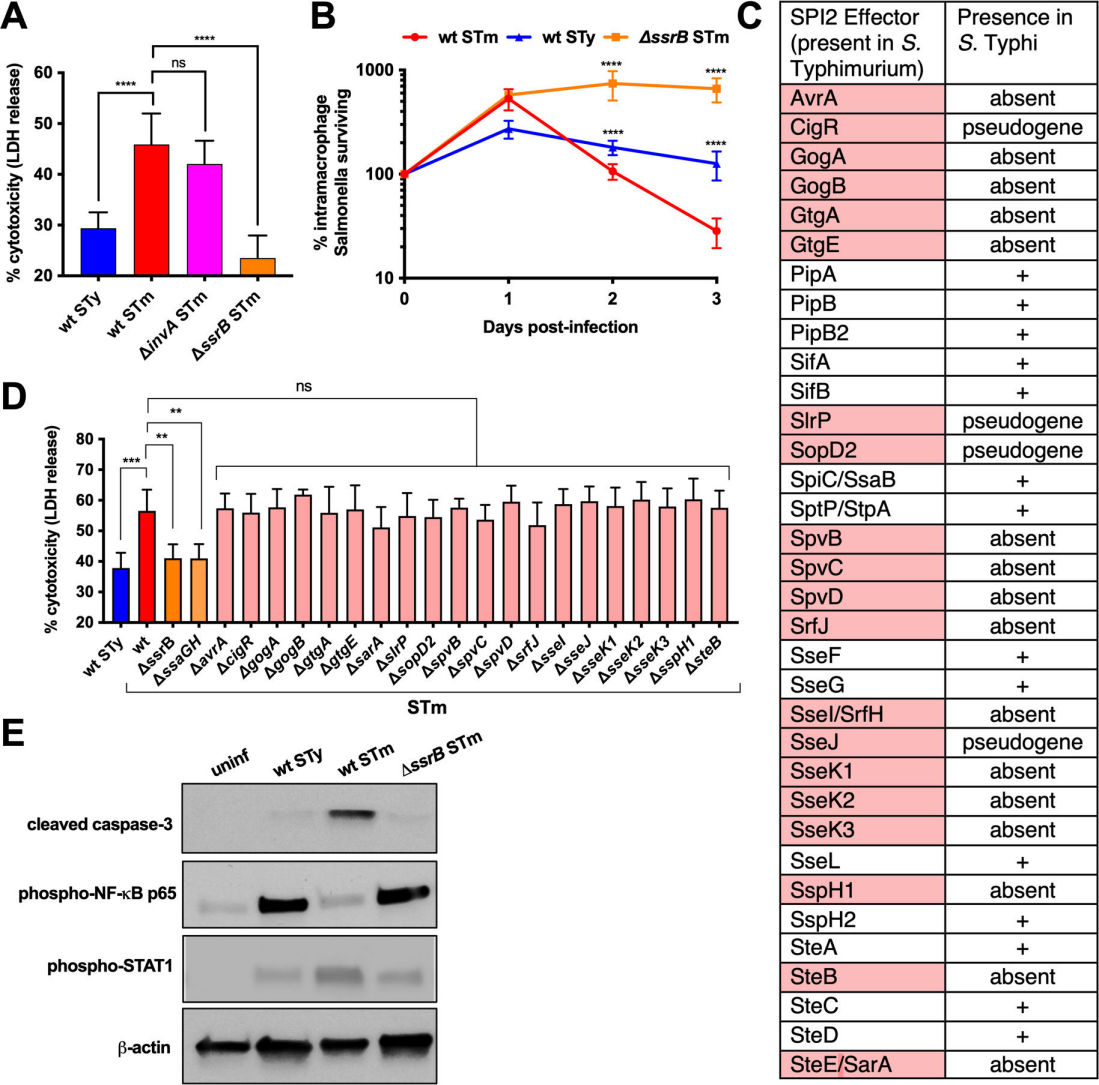
Differential cytotoxicity of *S.* Typhi and *S.* Typhimurium for human macrophages. THP-1 cells were differentiated with PMA and infected with opsonized stationary-phase *Salmonella* at a multiplicity of infection (MOI) of 10:1. *S*. Typhi Ty2 = STy, *S*. Typhimurium 14028s = STm. (**A**) Macrophage cytotoxicity was measured as the amount of LDH released in supernatants 24 hpi with wild-type or isogenic mutant *Salmonella* strains, *n* = 3. (**B**) Macrophages were lysed at the indicated time post-infection and intracellular colony-forming units (CFU) enumerated. Survival is expressed as the proportion of intracellular *Salmonella* remaining compared to internalized bacteria 1 hpi. Shown are the means and standard deviations of three separate experiments. (**C**) Table of effector proteins secreted by the SPI2-encoded T3SS in *S*. Typhimurium, with effectors that are absent or pseudogenes in *S*. Typhi indicated in red. (**D**) LDH released in supernatants 24 hpi from macrophages infected with wild-type or isogenic mutant *Salmonella* strains, *n* = 3. (**E**) Fifty micrograms of total protein from THP-1 cells infected with *Salmonella* for 24 h was subjected to western blot analysis for cleaved caspase-3, phosphorylated p65 subunit of NF-κB, or phosphorylated STAT1, with measurement of β-actin included as a loading control. Results from one representative experiment of three are shown. Bar graphs represent the means of three separate experiments with error bars representing standard deviations. Statistical significance was determined by one-way analysis of variance (ANOVA) with Tukey’s multiple comparisons (**A**) or Sidak’s multiple comparisons (**D**), or two-way ANOVA with Tukey’s multiple comparisons (**B**); ns, not significant; **P ≤ 0.0021; ***P ≤ 0.0002; ****P ≤ 0.0001.

*Salmonella* can spread from the intestinal tract to systemic sites within host phagocytic cells (22–25). We hypothesized that the ability of *S*. Typhi to cause persistent infection in humans is linked to its ability to sustain survival of infected macrophages. THP-1 monocyte-derived and human peripheral blood mononuclear cell (PBMC)-derived macrophages infected with *S*. Typhi exhibited less cytotoxicity than those infected with *S*. Typhimurium at 24 h post-infection (hpi) as measured by the release of lactate dehydrogenase (LDH) (Fig. 1A; Fig. S1A). Infection with *S*. Typhimurium T3SS mutants showed that macrophage LDH release is dependent on SPI2 (response regulator Δ*ssrB*) but not SPI1 (secretion apparatus component Δ*invA*) (Fig. 1A). The lack of cell death caused by an *S*. Typhimurium *ssrB* mutant correlates with its ability to persist and replicate in human macrophages (Fig. 1B).

While early studies of the nontyphoidal *S*. Typhimurium murine infection model demonstrated the importance of SPI2 for survival in macrophages and systemic infection in mice (10, 11), recent studies have suggested differences in the contribution of SPI2 to the virulence of NTS and enteric fever *Salmonella* (7, 26). *S*. Typhi is missing 20 out of 34 effectors secreted by SPI2 in *S*. Typhimurium, largely as a result of genomic decay (4) (Fig. 1C). Construction of *S*. Typhimurium strains lacking individual SPI2 effectors that are missing from *S*. Typhi did not identify a mutant associated with reduced LDH release from infected macrophages (Fig. 1D), but host pathways may be redundantly targeted by multiple SPI2 effectors. To examine the effect of *Salmonella* infection on various pathways, including caspase activation, JAK/STAT signaling, and transcriptional regulation, markers were assayed by western blot 24 hpi. *S*. Typhimurium infection induced activation of caspase-3, the executioner caspase of apoptosis, and sustained phosphorylation of the signaling molecule STAT1, an important regulator of macrophage activation and cell death (27–30) (Fig. 1E; Fig. S1B), whereas *S*. Typhi infection failed to activate these pathways. The transcription factor NF-κB promotes cell survival by inhibiting apoptotic factors such as Bcl-2 (31, 32). *S*. Typhimurium infection was not accompanied by activation of NF-κB p65, whereas *S*. Typhi infection resulted in NF-κB activation (Fig. 1E). Macrophages infected with an *S*. Typhimurium *ssrB* mutant showed that modulation of these three pathways is dependent on SPI2 (Fig. 1E; Fig. S1B). Differences in activated proteins were not due to differences in total protein levels between infection conditions (Fig. S1C through E). Therefore, the inability of *S*. Typhimurium to persist in human macrophages is attributable to SPI2-induced cytotoxicity, and conversely, *S*. Typhi persists within human macrophages by avoiding the activation of pathways leading to cell death.

### Promotion of macrophage cell death by the *S*. Typhimurium SPI2 effector SpvB

**Fig 2:**
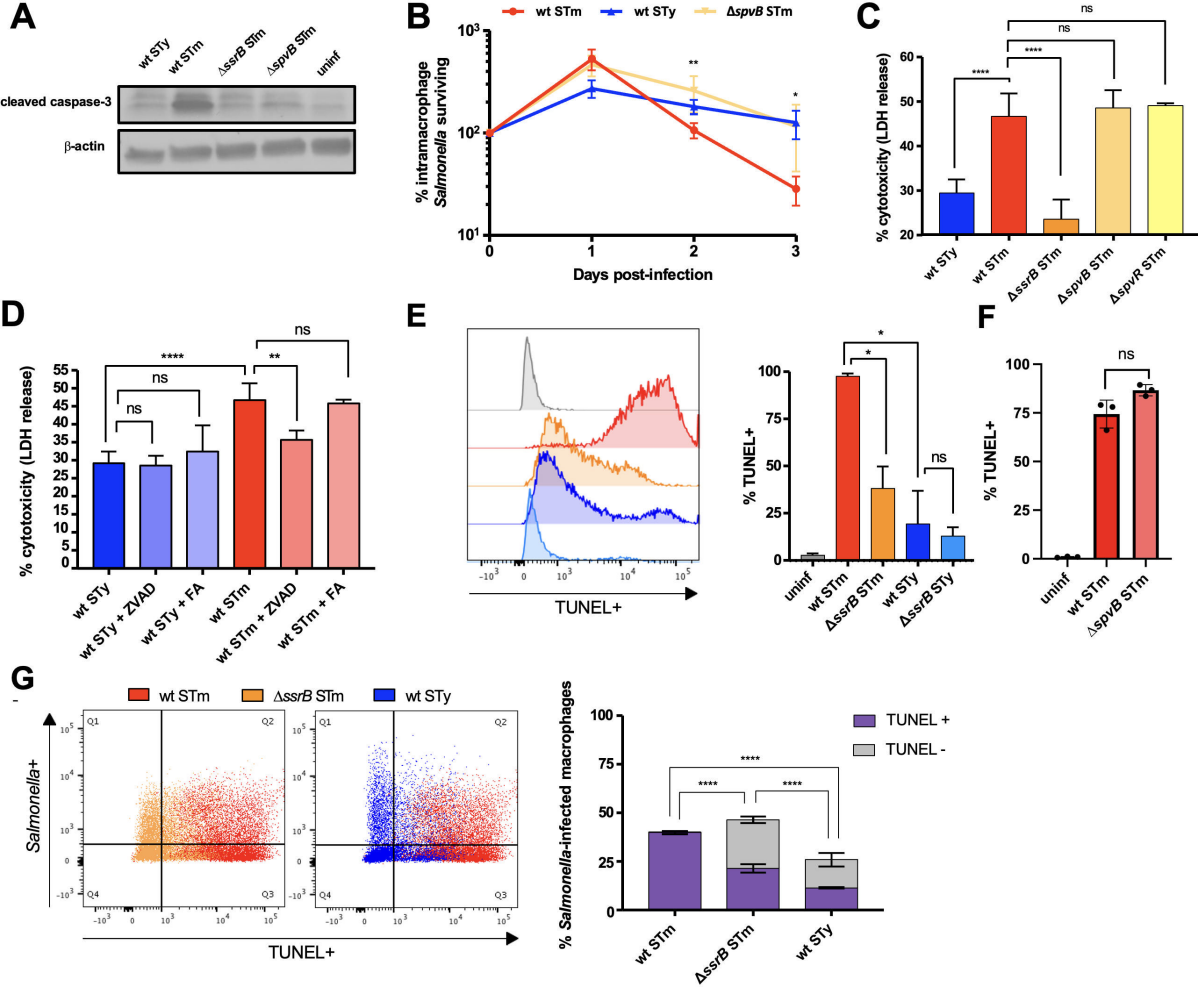
Apoptosis of *S.* Typhimurium-infected human macrophages is SPI2 dependent. THP-1 cells were differentiated with PMA and infected with opsonized stationary-phase *Salmonella* at an MOI of 10:1. *S*. Typhi Ty2 = STy, *S*. Typhimurium 14028s = STm. (**A**) Fifty micrograms of total protein from THP-1 cells infected with *Salmonella* for 24 h was subjected to western blot analysis for cleaved caspase-3. Activation of caspase-3 is dependent on the effector SpvB, which is absent in *S*. Typhi. Measurement of β-actin included as a loading control. Results from one representative experiment of three are shown. (**B**) Macrophages were lysed at the indicated time post-infection and intracellular CFU enumerated. Survival is expressed as the proportion of intracellular *Salmonella* remaining compared to internalized bacteria 1 hpi. Data for wild-type *S*. Typhimurium and *S*. Typhi provided for comparison are the same as in Fig. 1B. The means and standard deviations of three separate experiments are shown. (**C**) Macrophage cytotoxicity was measured as the amount of LDH released in supernatants 24 hpi with indicated strains, *n* = 3. (**D**) THP-1 cells were treated with 20 µM of the pan-caspase inhibitor Z-VAD-FMK (ZVAD) or the non-specific control molecule Z-FA-FMK (FA) 2 h prior to infection with *Salmonella*. LDH release was measured 24 hpi, *n* = 3. (**E**) *Salmonella*-infected macrophages were harvested 24 hpi and stained for terminal deoxynucleotidyl transferase dUTP nick-end labeling (TUNEL) as a measure of apoptosis. The left panel shows histograms from one representative experiment with TUNEL positivity on the *x*-axis. The right panel summarizes the means of three separate experiments. (**F**) TUNEL positivity was measured 24 hpi in macrophages infected with wild-type or *spvB* mutant *S*. Typhimurium. (**G**) *Salmonella* expressing the fluorochrome Ypet was used to infect macrophages and determine the population of infected cells during TUNEL analysis 24 hpi. The left panels show representative scatter plots with TUNEL positivity on the *x*-axis and *Salmonella* positivity on the *y*-axis. The right panel summarizes the means of three separate experiments. Bar graphs represent the means of three separate experiments with error bars representing standard deviations. Statistical significance *P* was determined by one-way ANOVA with Tukey’s multiple comparisons (**C**) or Sidak’s multiple comparisons (**D, E**), Student’s t test (**F**), or two-way ANOVA with Tukey’s multiple comparisons (**B, G**); ns, not significant; *P ≤ 0.0332; **P ≤ 0.0021; ***P ≤ 0.0002; ****P ≤ 0.0001.

*S*. Typhimurium mutant strains lacking individual SPI2 effectors were screened for their ability to activate caspase-3 in infected THP-1-derived human macrophages. The absence of the effector SpvB, encoded by the pSLT virulence plasmid and previously implicated in apoptosis (33–35), was sufficient to abrogate caspase-3 cleavage (Fig. 2A). SpvB is an ADP-ribosyl transferase that prevents the formation of F-actin and has been previously implicated in caspase-3 cleavage (36). SpvC, which has phosphothreonine lyase activity and inactivates MAP kinase (37), and SpvD, a cysteine protease that inhibits NF-κB signaling (38), were not required for caspase-3 cleavage. The *S*. Typhimurium *spvB* mutant also exhibited greater persistence within THP-1 macrophages (Fig. 2B). However, neither an *spvB* mutant nor an *spvR* mutant lacking the plasmid virulence regulator demonstrated reduced levels of macrophage LDH release 24 hpi (Fig. 2C), suggesting that *S*. Typhimurium promotes macrophage cytotoxicity by both caspase-3-dependent and -independent mechanisms. Indeed, a pan-caspase inhibitor reduced, but did not completely abrogate, cell death in *S*. Typhimurium-infected cells (Fig. 2D). The inhibitor had no effect on LDH release from *S*. Typhi-infected cells, indicating these cells were not undergoing apoptosis (Fig. 2D).

### Absence of apoptosis in *S*. Typhi-infected human macrophages

As the experimental conditions were selected to minimize SPI1-dependent pyroptosis (27), and caspase-3 cleavage was detected, we hypothesized that *S*. Typhimurium-induced cell death is primarily due to apoptosis. Infected THP-1 macrophages were assayed by TUNEL staining to detect DNA fragmentation as a measurement of apoptosis. *S*. Typhimurium infection resulted in ~97% TUNEL positivity of infected macrophages, and an *ssrB* mutant strain exhibited a 60% reduction in TUNEL positivity (Fig. 2E). Macrophages infected with either wild-type or *ssrB* mutant *S*. Typhi also exhibited significantly lower levels of TUNEL positivity (Fig. 2E), but apoptosis was not dependent on the presence of *spvB* (Fig. 2F). To determine whether TUNEL-positive cells contained bacteria, *Salmonella* expressing the fluorochrome Ypet was used for infection. Although a higher proportion of macrophages contained *S*. Typhimurium in comparison to *S*. Typhi, restricting the analysis to macrophages containing *Salmonella* showed that *S*. Typhimurium-infected cells exhibit higher levels of TUNEL positivity than those infected with *S*. Typhi (Fig. 2G). Stratification of macrophages on the basis of Ypet fluorescence intensity demonstrated that TUNEL positivity was related to serovar, but not organism, load (Fig. S2). Bystander apoptosis of uninfected cells was also observed during wild-type infection with *S*. Typhimurium, a phenomenon observed in association with other intracellular pathogens (39, 40). An *S*. Typhimurium *ssrB* mutant was able to infect a high proportion of macrophages but led to significantly less TUNEL positivity (Fig. 2G). Thus, macrophage infection with *S*. Typhimurium rapidly induces SPI2-dependent DNA fragmentation indicative of apoptosis, whereas *S*. Typhi fails to induce this response.

### Absence of apoptosis in *S*. Paratyphi-infected human macrophages

**Fig 3:**
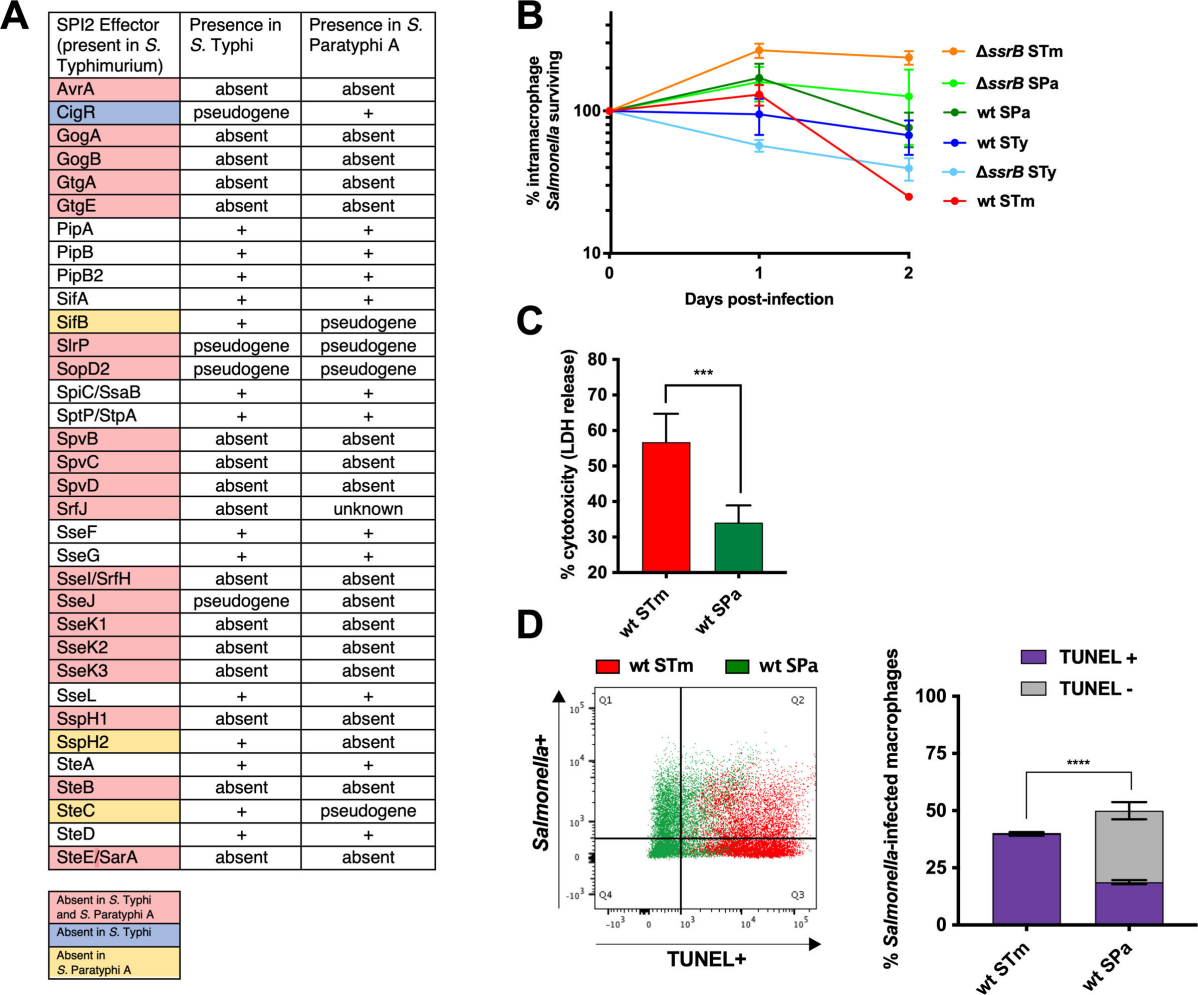
Human macrophages infected with *S.* Paratyphi A do not exhibit SPI2-dependent apoptosis. THP-1 cells were differentiated with PMA and infected with opsonized stationary-phase *Salmonella* at an MOI of 10:1. *S*. Typhimurium 14028s = STm, *S*. Typhi Ty2 = STy, *S*. Paratyphi A ATCC9150 = SPa. (**A**) Table of effector proteins secreted by SPI2 in *S*. Typhimurium, with effectors that are absent or pseudogenes in *S*. Typhi and *S*. Paratyphi A are indicated in red. (**B**) Macrophages were lysed at indicated times post-infection and intracellular CFU enumerated. Survival is expressed as the proportion of intracellular *Salmonella* remaining compared to internalized bacteria 1 hpi. The means of three biological replicates are shown. (**C**) Macrophage cytotoxicity was measured as the amount of LDH released in supernatants 24 hpi with indicated strains. (**D**) *Salmonella* expressing the fluorochrome Ypet was used to infect macrophages and determine the population of infected cells during TUNEL analysis 24 hpi. The left panels show representative scatter plots with TUNEL positivity on the *x*-axis and *Salmonella* positivity on the *y*-axis. Data for *S*. Typhimurium provided for comparison are identical to what is shown in Fig. 2G. The right panel summarizes the means of three separate experiments. Bar graphs represent the means of three separate experiments with error bars representing standard deviations. Statistical significance was determined by paired two-tailed Student’s *t*-test (**C**) or two-way ANOVA with Tukey’s multiple comparisons (**D**); ***P ≤ 0.0002; ****P ≤ 0.0001.

*Salmonella* Paratyphi A is a human-restricted serovar that causes a clinical syndrome indistinguishable from typhoid fever and has undergone genomic decay resulting in loss of the same 12 pro-apoptotic SPI2 effectors that are absent from *S*. Typhi (Fig. 3A). We hypothesized that similar interactions with human macrophages might account for the similar clinical features of *S*. Typhi and *S*. Paratyphi A infections. In support of this hypothesis, both *S*. Paratyphi A and *S*. Typhi persisted within THP-1-derived macrophages at 48 hpi (Fig. 3B). LDH release was lower in *S*. Paratyphi-infected macrophages at 24 h (Fig. 3C), along with reduced evidence of apoptosis (Fig. 3D), in comparison to *S*. Typhimurium-infected cells. As with *S*. Typhi, TUNEL positivity was not dependent on organism load (Fig. S2). These observations suggest that *S*. Typhi and *S*. Paratyphi A have convergently evolved the ability to persist within human macrophages due to the loss or absence of pro-apoptotic SPI2 effectors.

### Inhibition of NF-κB activation during *S*. Typhimurium infection

**Fig 4:**
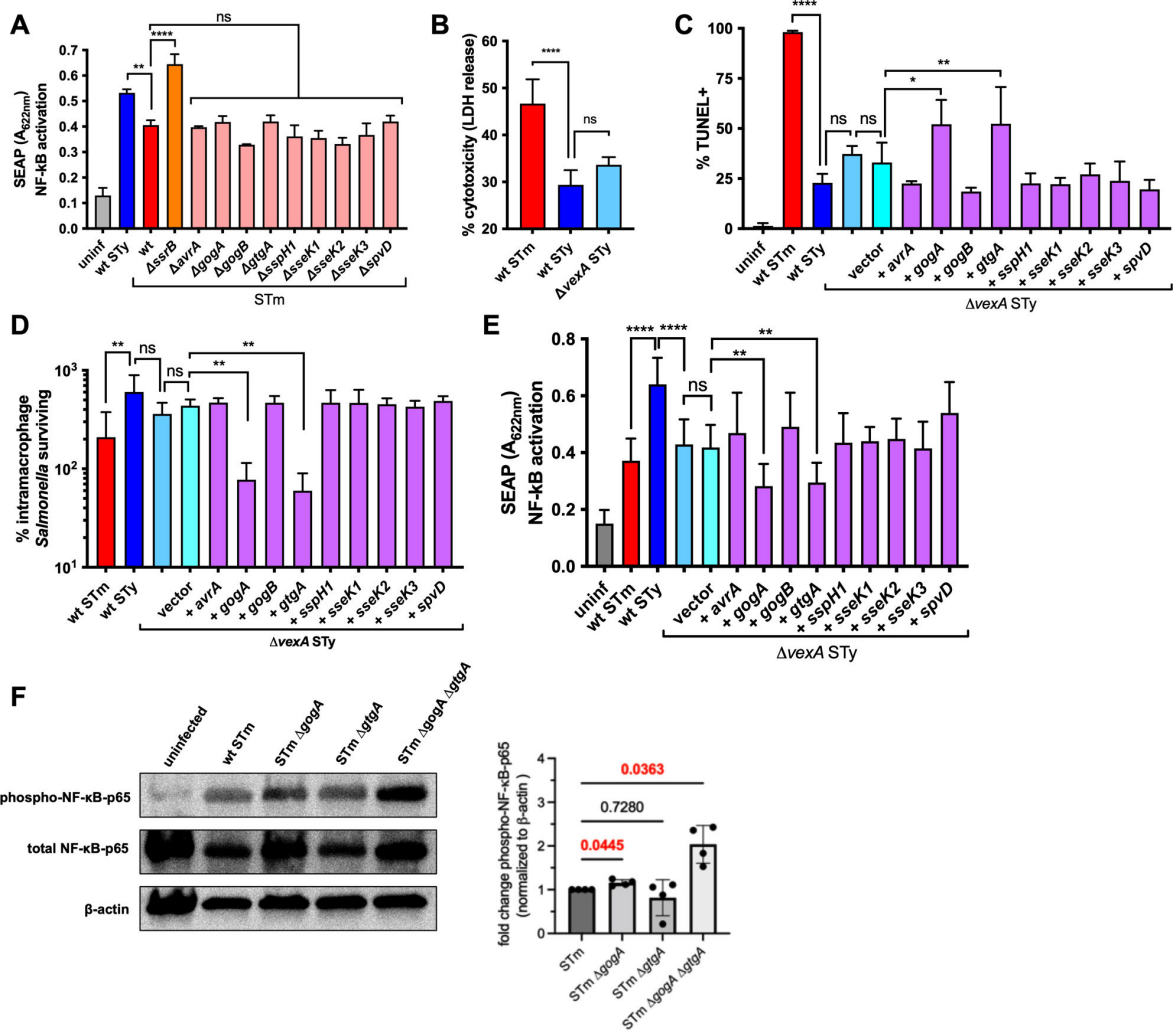
SPI2 effectors inhibit NF-κB activation during *S.* Typhimurium infection. THP-1 cells were differentiated with PMA and infected with opsonized stationary-phase *Salmonella* at an MOI of 10:1. *S*. Typhimurium 14028s = STm, *S*. Typhi Ty2 = STy. (**A**) THP-1 NF-κB Blue reporter cells were infected with *Salmonella* and NF-κB activation measured 24 hpi using the colorimetric Quanti-Blue assay. (**B**) Macrophage cytotoxicity was measured as the amount of LDH released in supernatants 24 hpi with indicated strains. (**C**) *Salmonella*-infected macrophages were harvested 24 hpi and stained for TUNEL as a measure of apoptosis. (**D**) Macrophages were lysed at 1 or 24 hpi and intracellular CFU enumerated. Survival is expressed as the proportion of intracellular *Salmonella* remaining compared to internalized bacteria 1 hpi. (**E**) THP-1 NF-κB Blue reporter cells were infected with *Salmonella* and NF-κB activation measured 24 hpi using the colorimetric Quanti-Blue assay. SPI2 effector genes in (**C–E)** were expressed from a plasmid. (**F**) Fifty micrograms of total protein from THP-1 cells infected with *Salmonella* for 24 h was subjected to western blot analysis for activated NF-κB p65, with measurement of β-actin included as a loading control. Results from one representative experiment of four are shown on the left, with summary data on the right. Bar graphs represent the means of three separate experiments with error bars representing standard deviations. Statistical significance was determined by one-way ANOVA with Sidak’s multiple comparisons (**A–E**) or Dunnett’s multiple comparisons test (**F**); ns, not significant; *P ≤ 0.0332; **P ≤ 0.0021; ***P ≤ 0.0002; ****P ≤ 0.0001.

Nine of the SPI2 effectors that are present in *S*. Typhimurium but absent in *S*. Typhi and *S*. Paratyphi A have been specifically implicated in the inhibition of the NF-κB regulatory complex (38, 41–46). Individual effector mutants of *S*. Typhimurium were screened for their ability to activate NF-κB p65 using a THP-1 Blue reporter cell line in which cells express secreted alkaline phosphatase (SEAP) enzyme under the control of an NF-κB-activated promoter (Invivogen) (Fig. 4A). None of the *S*. Typhimurium mutant strains lacking individual SPI2 effectors activated NF-κB above wild-type levels, in contrast to an *ssrB* mutant, suggesting that some effectors have redundant effects on the NF-κB pathway. Therefore, as an alternative approach, each individual effector was heterologously expressed in *S*. Typhi to examine its effect on macrophage apoptosis. As the Vi capsule reduces levels of *Salmonella* internalization, effector complementation was carried out in a Vi mutant *S*. Typhi background to allow levels of bacterial internalization similar to that of *S*. Typhimurium (47). Absence of the Vi capsule did not affect macrophage cytotoxicity (Fig. 4B). Infected macrophages were assayed for apoptosis by TUNEL staining, and two of the heterologously expressed effectors, GogA and GtgA, were able to increase TUNEL positivity in *S*. Typhi-infected macrophages (Fig. 4C). *S*. Typhi strains containing GogA or GtgA were similarly attenuated in their ability to persist in macrophages 24 hpi (Fig. 4D). Infection of THP-1 Blue reporter cells demonstrated that both GogA and GtgA were able to reduce NF-κB activation when expressed in *S*. Typhi (Fig. 4E). A GogA/GtgA double mutant *S*. Typhimurium strain induced a significant increase in activated NF-κB in infected macrophages (Fig. 4F). The SseK SPI2 effectors also have redundant functions, but an SseK1/SseK2/SseK3 triple mutant was similar to single mutants or wild-type *S*. Typhimurium in the degree of cytotoxicity and NF-κB activity observed in infected macrophages (Fig. S3A and B).

### Apoptosis of *S*. Typhi-infected macrophages following pharmacologic NF-κB inhibition

**Fig 5:**
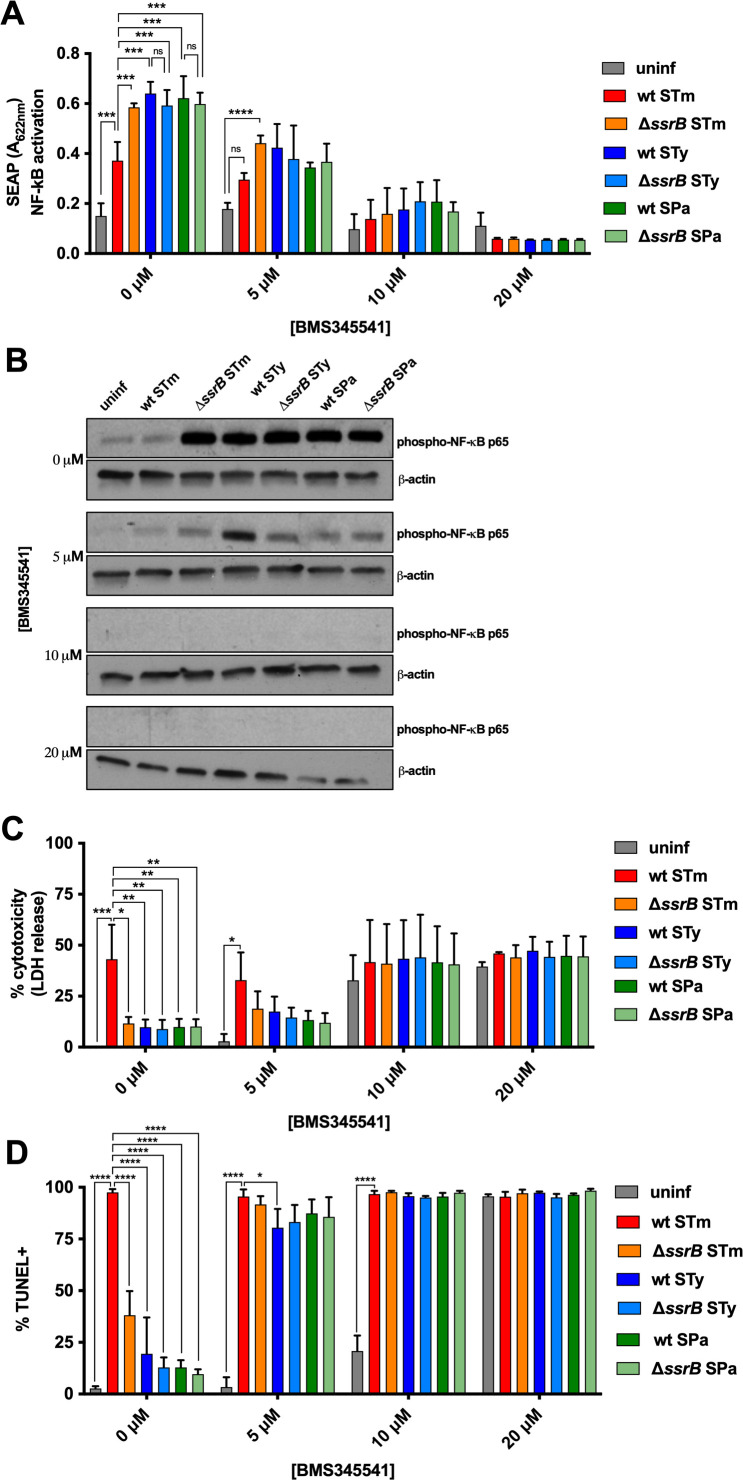
Pharmacologic inhibition of NF-κB during *Salmonella* infection results in apoptosis of infected macrophages. THP-1 cells were differentiated with PMA and infected with opsonized stationary-phase *Salmonella* at an MOI of 10:1. Macrophages were treated with the indicated concentration of the NF-κB inhibitor BMS345541 1 h prior to infection and treatment maintained throughout the infection. *S*. Typhimurium 14028s = STm, *S*. Typhi Ty2 = STy, *S*. Paratyphi A ATCC9150 = SPa. (**A**) THP-1 NF-κB Blue reporter cells were infected with *Salmonella* and NF-κB activation measured 24 hpi using the colorimetric Quanti-Blue assay. (**B**) Fifty micrograms of total protein from THP-1 cells infected with *Salmonella* for 24 h was subjected to western blot analysis for activated NF-κB p65, with measurement of β-actin included as a loading control. Results from one representative experiment of three are shown. (**C**) Macrophage cytotoxicity was measured as the amount of LDH released in supernatants 24 hpi with the indicated strains. (**D**) Macrophages were stained for TUNEL 24 h after *Salmonella* infection. Bar graphs represent the means of three separate experiments with error bars representing standard deviations. Statistical significance was determined by two-way ANOVA with Dunnett’s multiple comparison test; *P ≤ 0.0332; **P ≤ 0.0021; ***P ≤ 0.002; ****P ≤ 0.0001.

To confirm the importance of NF-κB activation in determining macrophage cell fate during *Salmonella* infection, infected cells were treated with the pharmacologic NF-κB inhibitor BMS345541 (Cayman Chemical). BMS345541 treatment resulted in dose-dependent inhibition of NF-κB activation in macrophages infected with either wild-type or *ssrB* mutant derivatives of *S*. Typhimurium, *S*. Typhi, or *S*. Paratyphi A, as measured in the THP-1 Blue reporter cell line (Fig. 5A). Increased phosphorylation of NF-κB p65 was observed in macrophages infected with *ssrB* mutant *S*. Typhimurium, *S*. Typhi, or *S*. Paratyphi A, but not in macrophages infected with wild-type *S*. Typhimurium (Fig. 5B). Treatment with BMS345541 inhibited p65 phosphorylation in all infected macrophages, even at low doses (Fig. 5B). Inhibition of NF-κB correlated with an increase in macrophage cell death as measured by LDH release (Fig. 5C) or by TUNEL staining (Fig. 5D; Fig. S4A). Low doses of BMS345541 or a different NF-κB inhibitor, TPCA-1, were sufficient to selectively induce TUNEL-positive apoptosis of *Salmonella*-infected cells (Fig. 5D; Fig. S4B and C), whereas only high doses of BMS345541 induced apoptosis of uninfected cells (Fig. 5D).

### Activation of STAT1 by the *S*. Typhimurium SPI2 effector SarA

**Fig 6:**
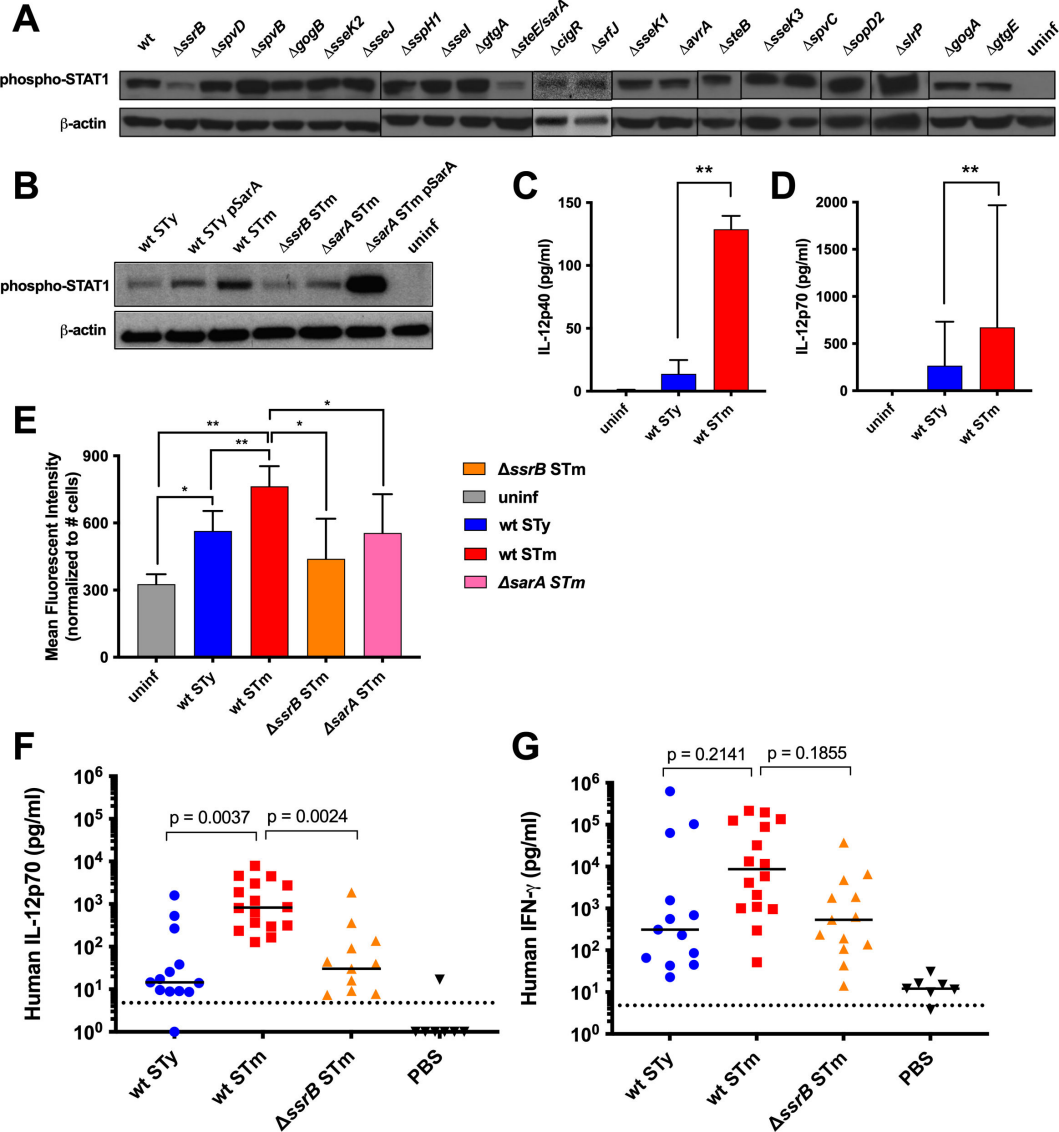
STAT1 activation and pro-inflammatory cytokine production in *Salmonella-*infected human macrophages and humanized mice are SPI2 dependent. THP-1- or PBMC-derived macrophages were infected with opsonized stationary-phase *Salmonella* at an MOI of 10:1. *S*. Typhimurium 14028s = STm, *S*. Typhi Ty2 = STy. (**A, B**) Fifty micrograms of total protein from THP-1 cells infected with *Salmonella* for 24 h was subjected to western blot analysis for phosphorylated STAT1, with measurement of β-actin included as a loading control. Results from one representative experiment of three are shown. (**C, D**) Culture supernatants from *Salmonella*-infected THP-1 cells (**C**) or PBMC (**D**) were assayed for the presence of human IL-12 by enzyme-linked immunosorbent assay (ELISA) 24 hpi, *n* = 3. (**E**) Infected PBMC-derived macrophages were treated with Brefeldin A and stained for intracellular retention of IL-12p70 24 hpi. Bar graphs represent the means of three separate experiments with error bars representing standard deviations. Statistical significance was determined by paired two-tailed Student’s *t*-test; *P ≤ 0.05; **P ≤ 0.01. (**F, G**) Humanized CD34+Hu NSG mice were infected intraperitoneally with *Salmonella* and analyzed for human cytokines IL-12p70 (**F**) or IFNγ (**G**) by multiplex microbead cytokine array (Luminex). Dotted line indicates assay limit of detection. Statistical significance was determined by paired two-tailed Student’s *t*-test (**C, D**), one-way ANOVA with Sidak’s multiple comparisons (**E**), or Kruskal–Wallis test (**F, G**); *P ≤ 0.0332; **P ≤ 0.0021.

The eukaryotic transcription factor STAT1 promotes macrophage polarization, activation of inflammatory responses, and expression of pro-apoptotic genes including tumor necrosis factor alpha (TNFα) and caspases (28, 48, 49). *S*. Typhimurium mutant strains lacking individual SPI2 effectors were screened for their ability to activate STAT1 in infected macrophages by western blot at 24 hpi. The absence of the effector SarA (also known as SteE) was sufficient to abrogate STAT1 phosphorylation in infected macrophages, similar to *ssrB* mutant *S*. Typhimurium (Fig. 6A) or wild-type *S*. Typhi (Fig. 1E and 6B). Measurement of phospho-STAT1 over time suggested that *S*. Typhi promotes STAT1 dephosphorylation (Fig. S5A). The addition of exogenous IFNβ to stimulate STAT1 activation did not raise levels in *S*. Typhi-infected macrophages to those observed in *S*. Typhimurium-infected cells (Fig. S5B), but treatment with IFNγ enhanced STAT1 phosphorylation during *S*. Typhi infection (Fig. 5C).

Although expression of genes encoding the STAT1-dependent cytokines CXCL10 and TNFα was not affected by *sarA* at 24 hpi (Fig. S5D and E), STAT1 dephosphorylation in the absence of *sarA* might influence downstream gene expression at later timepoints. Phosphorylation of STAT1 could be restored during infection with *sarA* mutant *S*. Typhimurium complemented with a *sarA*-expressing plasmid, and the heterologous expression of SarA in *S*. Typhi also increased levels of phosphorylated STAT1 (Fig. 6B), demonstrating that SarA is responsible for the sustained phosphorylation of STAT1 observed in *S*. Typhimurium-infected macrophages.

### Failure of *S.* Typhi to induce T_H_1 polarization in human macrophages and humanized mice

To determine whether *Salmonella-*modulated STAT1 phosphorylation affects T_H_1 immune responses, IL-12 production was measured in infected macrophages. THP-1- or human PBMC-derived macrophages were infected with either *S*. Typhi or *S*. Typhimurium and supernatants assayed by ELISA 24 hpi. *S*. Typhimurium-infected macrophages produced significantly higher levels of IL-12 than *S*. Typhi-infected macrophages (Fig. 6C and D). Due to differential macrophage cell death caused by *S*. Typhi and *S*. Typhimurium infection (Fig. 1A), measurement of cytokines in the supernatant may not account for differences in surviving macrophage numbers. Therefore, intracellular cytokine staining was performed to normalize the concentration of IL-12 to viable cell numbers. *S*. Typhi-infected macrophages produced significantly lower levels of IL-12p70 than *S*. Typhimurium when equal numbers of macrophages were compared (Fig. 6E). An *ssrB* mutant *S*. Typhimurium induced less IL-12 production, as did a *sarA* mutant strain, implicating STAT1 activation in the elicitation of T_H_1 cytokines by *S*. Typhimurium (Fig. 6E).

A humanized mouse model of acute septicemic typhoid fever was used to determine whether *S*. Typhimurium and *S*. Typhi infections differ in the induction of T_H_1 cytokine production *in vivo*. CD34 +Hu NSG mice are rendered susceptible to lethal *S*. Typhi infection due to the presence of functional human hematopoietic cells (50). CD34+Hu NSG mice were infected with wild-type *S*. Typhi, wild-type *S*. Typhimurium, or *ssrB* mutant *S*. Typhimurium by intraperitoneal inoculation, and serum was collected for cytokine analysis after mice were humanely euthanized. *S*. Typhimurium infection of humanized mice resulted in elevated levels of the human T_H_1 cytokines IL-12 (Fig. 6F) and IFNγ (Fig. 6G), whereas infection with *ssrB* mutant *S*. Typhimurium or *S*. Typhi elicited lower cytokine expression, with significantly lower levels of IL-12. Higher organ burdens in wild-type *S*. Typhimurium-infected mice (Fig. S6) may be a contributing factor. As the importance of a T_H_1 immune response for the clearance of NTS infections has been amply demonstrated (51, 52), the ability of *S*. Typhi to avoid T_H_1 polarization may contribute to its ability to cause persistent infection.

## DISCUSSION

**Fig 7:**
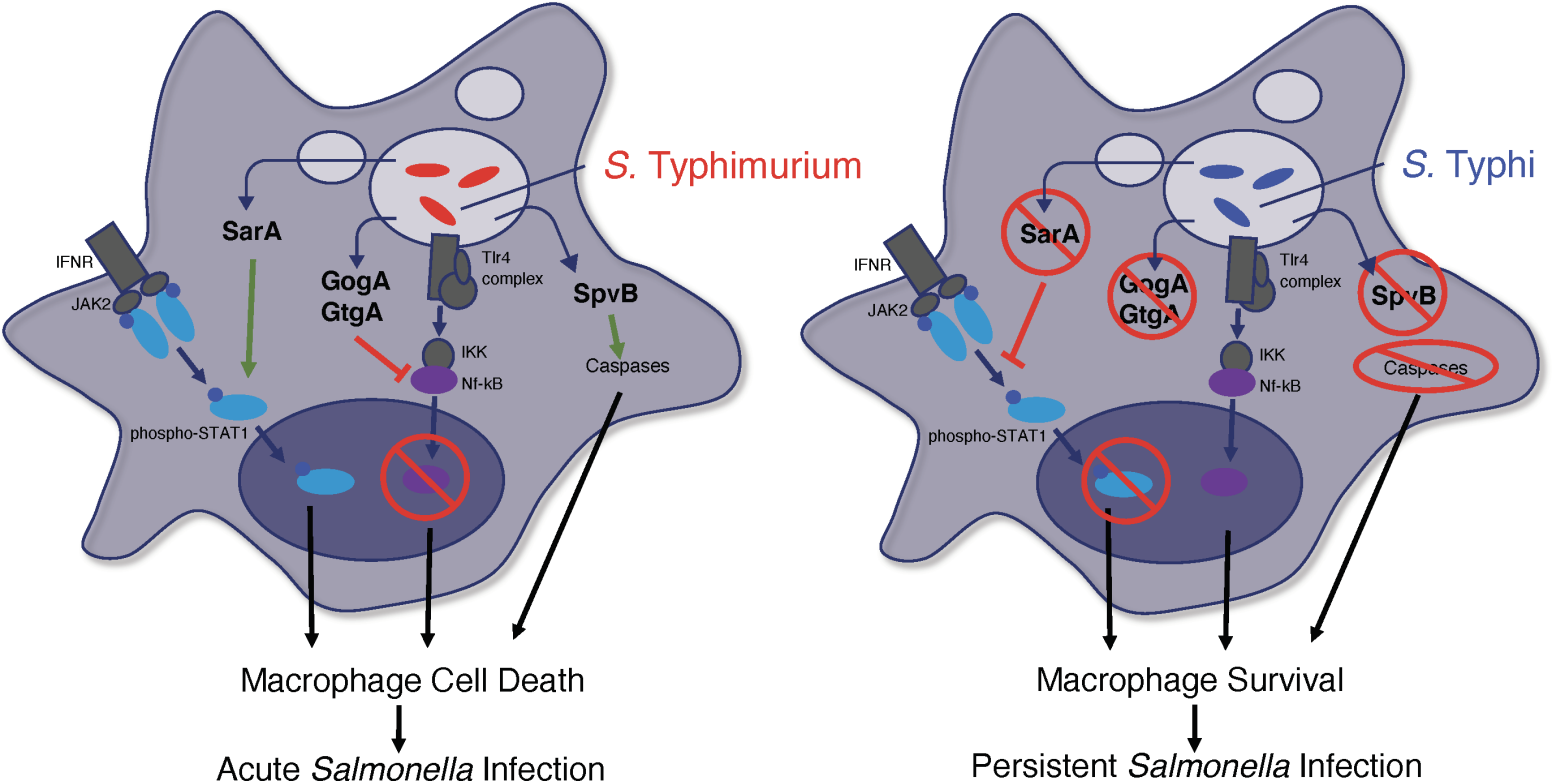
Model of infection of human macrophages with nontyphoidal (*S.* Typhimurium) and enteric fever (*S.* Typhi) *Salmonella* serovars. During *S*. Typhimurium infection of human macrophages, the presence of SPI2-secreted effectors, such as SarA, GogA, GtgA, and SpvB, leads to induction of cell death pathways, resulting in macrophage apoptosis and promoting acute inflammatory enteritis. Absence of these effectors in *S*. Typhi and *S*. Paratyphi A leads to macrophage survival and persistent infection characteristic of enteric fever.

Our observations demonstrate fundamental differences in the interaction of nontyphoidal and enteric fever *Salmonella* serovars with human macrophages. The enteric fever serovars *S*. Typhi and *S*. Paratyphi A grow and persist within human macrophages through the sustained expression of cell survival pathways regulated by NF-κB, whereas intracellular replication of *S*. Typhimurium is rapidly followed by macrophage apoptosis and the initiation of a T_H_1 cellular immune response (Fig. 7). A prolonged incubation period followed by systemic dissemination, sustained infection, and chronic carriage are hallmarks of enteric fever that reflect the ability of *S*. Typhi and *S*. Paratyphi A to persist within macrophages and avoid immune clearance (53, 54). The divergent interactions of nontyphoidal and enteric fever *Salmonella* serovars with human macrophages provide a mechanistic explanation for the divergent clinical features of enteritis and enteric fever.

The SPI2 pathogenicity island is required for virulence in the murine *S*. Typhimurium model, but accumulating evidence suggests that SPI2 plays a different role in the pathogenesis of enteric fever. SPI2 is not required for *in vitro* infection of human macrophages with *S*. Typhi (26) and is expressed at lower and more variable levels following macrophage internalization compared to *S*. Typhimurium (7, 26, 55). A comprehensive screen for virulence genes identified SPI2 during murine *S*. Typhimurium infection but not during *S*. Typhi infection of humanized mice (7). Here, we show that the loss of specific SPI2 effectors by *S*. Typhi and *S*. Paratyphi A allows these serovars to persistently infect human phagocytes.

Specific *S*. Typhimurium T3SS effectors interact with pyroptotic, apoptotic, and necroptotic pathways to induce host cell death and recruit inflammatory responses (56). Studies in murine macrophages have shown that caspase-1-dependent pyroptosis is rapidly triggered in a process dependent upon the SPI-1 T3SS and flagella (57), while delayed apoptosis is dependent on the SPI-2 T3SS (17). Human macrophages also exhibit early SPI-1-dependent cell death (58) and delayed SPI-2-dependent cell death (59). Delayed cytotoxicity in human macrophages infected with nontyphoidal *Salmonella enterica* serovar Dublin has been shown to be dependent on the SPI-2 effector SpvB, an ADP-ribosyltransferase that modifies host actin (35, 60), leading to caspase-3 activation (59). The present study confirms SPI-2-dependent apoptosis of *S*. Typhimurium-infected human macrophages and SpvB-dependent caspase-3 cleavage. However, under our conditions, elimination of SpvB was not sufficient to eliminate SPI-2-dependent cytotoxicity in *S*. Typhimurium-infected human macrophages, suggesting a contribution from caspase-3-independent apoptosis, as described for *M. tuberculosis* (61).

An important class of SPI2 effectors that is absent from enteric fever *Salmonella* serovars comprises the nine effectors that inhibit NF-κB. Here, we show that the zinc metalloproteases GogA and GtgA play prominent roles in NF-κB inactivation, consistent with their ability to cleave the p65, RelB, and cRel components of NF-κB (45, 62). Notably, other intracellular pathogens have been shown to activate NF-κB to promote host cell survival, including *Mycobacterium tuberculosis* and *Legionella pneumophila* (63–66). We show that pharmacologic inhibition of NF-κB is sufficient to induce selective apoptosis of *S*. Typhi-infected macrophages without affecting uninfected cells, suggesting that NF-κB inhibition might provide the basis for a novel therapeutic approach to eliminate persistent intracellular pathogens that utilize a similar strategy.

The present study also shows that the sustained phosphorylation of STAT1 and subsequent expression of the T_H_1 cytokine IL-12 are dependent on the SPI2 effector SarA, which is absent in *S*. Typhi and *S*. Paratyphi A. Pathogen-induced IL-12 production plays a central role in the initiation of T_H_1 polarization (67) and downstream T-cell activation (68). SarA has been previously shown to promote STAT3 phosphorylation, leading to M2 polarization of Salmonella-infected macrophages (69–72), indicating that SarA can potentiate both STAT1- and STAT3-dependent immune signaling. This suggests that the effects of SarA on macrophage polarization may be dependent on additional factors. *S*. Typhi and *S*. Typhimurium exhibit fundamental differences with regard to T_H_1 immune responses. Individuals with inherited or acquired defects in T_H_1 immunity, such as those with IFNγ/IL12B/IFNγR1 polymorphisms or HIV/AIDS, are more susceptible to nontyphoidal *Salmonella* infections, but not to enteric fever (19, 52, 73). Expression of IL-12 is associated with initiation of a T_H_1 response and stimulates other immune cells to produce IFNγ, which drives macrophages toward an M1 phenotype. Thus, the contribution of SarA to a T_H_1 response may be context dependent.

Although the murine typhoid model has revealed important insights into shared features of *Salmonella* pathogenesis, reliance on this model may have delayed an appreciation of the unique aspects of *S*. Typhi and *S*. Paratyphi A virulence. The present study adds to previous observations in *Shigella flexneri* and *Yersinia pestis* (74, 75) by providing an example of the evolution of virulence by gene loss. Genomic decay manifested as gene deletion or pseudogene formation is observed in certain pathogens that have developed increasing host specialization, such as *Yersinia pestis*, *Mycobacterium leprae*, and *Salmonella* serovars including *S*. Gallinarum, *S*. Dublin, *S*. Typhi, and *S*. Paratyphi A (76–79). Although most genomic decay is attributable to the loss of functions that are no longer required by bacteria following adaptation to a specialized lifestyle (3), pathoadaptive gene loss has also been described (80). Here, we provide evidence that the loss of specific SPI-2 T3SS effectors has endowed *S*. Typhi and *S*. Paratyphi A with the ability to persist within human macrophages without inducing cell death, thereby enabling the establishment of a novel host–pathogen relationship.

Our observations reveal a fundamental divergence between nontyphoidal *Salmonella*, which induces macrophage apoptosis and exploits the host inflammatory response, and enteric fever *Salmonella* serovars, which avoid the induction of T_H_1 immunity and persist in an intracellular compartment by promoting macrophage survival. Whereas macrophage apoptosis plays a central role in the pathogenesis of nontyphoidal *Salmonella* infections, the prevention of apoptosis is a conserved feature of *Salmonella* serovars that cause enteric fever and has important implications for the treatment of chronic bacterial infections.

## MATERIALS AND METHODS

### Bacterial growth conditions and strain constructions

Bacterial strains and plasmids used in this study are listed in Table S1. *S. enterica* cultures were grown in Miller’s Luria broth (LB) medium at 37°C with shaking at 250 rpm. The medium was supplemented with “aromix” (40 µg mL^−1^ of L-phenylalanine, 40 µg mL^−1^ of L-tryptophan, 10 µg mL^−1^ of 2,3-dihydroxybenzoic acid, and 10 µg mL^−1^ of p-amino benzoic acid), and ampicillin (100 µg mL^−1^) or kanamycin (50 µg mL^−1^), as indicated.

Primers were purchased from Integrated DNA Technologies (IDT, Skokie, IL) and are listed in Table S2. Mutant alleles of *S. enterica* serovars were constructed using λ-Red recombination as described (81, 82). All PCR products were generated with gDNA from *S*. Typhimurium 14028s or *S*. Typhi Ty2. All mutant strains and plasmid constructs were confirmed by DNA sequencing (Genewiz, South Plainfield, NJ).

To construct the SarA complementation plasmid, the *sarA* gene and promoter region were amplified from *S*. Typhimurium gDNA using primers listed in Table S2 and ligated into pJK392 digested with KpnI and HindIII. To construct a plasmid containing *gogA*, *sarA*, and *pagK2*, a 5-kb region described as unique to strains that produce IL-10 (70) was amplified from *S*. Typhimurium gDNA using primers listed in Table S2 and ligated into the SpeI and ApaI sites of pWSK129. To construct SPI2 effector complementation plasmids pJK761, pJK762, pJK764, pJK766, pJK767, pJK768, and pJK769, a constitutive promoter region was amplified using pDNA from pTrc99a, and the effector gene was amplified from *S*. Typhimurium gDNA using primers listed in Table S2. The two fragments were assembled using NEBuilder HiFi Assembly Master Mix (NEB, Ipswich, MA), PCR amplified, then assembled in a second HiFi Assembly reaction with pRB3-273C digested with BamHI. To construct SPI2 effector complementation plasmids pJK763 and pJK765, the effector gene was amplified from *S*. Typhimurium gDNA and assembled with pJK770 digested with NcoI using HiFi Assembly. The empty SPI2 effector expression plasmid pJK724 was constructed by assembling the promoter from pTrc99a pDNA with pRB3-273C digested with PstI and HindIII using HiFi Assembly.

The *S*. Typhimurium *gogA/gtgA* double mutant was constructed by transduction using phage P22 HT105/1 *int*-201. One hundred microliters of stationary-phase culture of the recipient strain was mixed with 1 µL of P22 lysate. After 1 h of incubation at 37°C, transductants were selected on LB agar plates with antibiotic selection for the appropriate selectable marker. Colonies were streaked on indicator Green Plates, and P22-free colonies were selected for further experiments.

### THP-1 macrophage cell culture and infection

Human THP-1 monocytes were obtained from ATCC and cultured in RPMI 1640 medium (Corning Inc.) supplemented with 10% heat-inactivated fetal bovine serum (Millipore-Sigma), sodium pyruvate (Corning Inc.), non-essential amino acids (Gibco), 50 U mL^−1^ of penicillin and 50 µg mL^−1^ of streptomycin (Corning Inc.) at 37°C in 5% CO2.

Human THP-1-derived macrophages were infected as described previously (7). Briefly, THP-1 monocytes were seeded at 10^5^ per well in 96-well plates or 5 × 10^5^ in 24-well plates and differentiated with 100 nM of phorbol 12-myristate 13-acetate (PMA; Millipore-Sigma) for 48 h; the medium was changed to PMA-minus and antibiotic-free RPMI 24 h prior to infection. *Salmonella* strains were grown in LB broth for 18 h with shaking at 37°C, then adjusted to OD_600_ = 1.0 and washed twice with sterile phosphate-buffered saline (PBS). *Salmonella* was mixed with equal parts of human pooled serum (MP Biomedicals LLC) and incubated at 37°C for 20 min to opsonize bacteria. Opsonized bacteria were used to infect THP-1 human macrophage-like cells at an MOI of 10:1. Infected monolayers were centrifuged for 5 min at 1,000 rpm to synchronize infection, then incubated at 37°C for 1 h to promote internalization. Following internalization, monolayers were washed with RPMI supplemented with 20 µg mL^−1^ of gentamicin to kill extracellular bacteria.

For infections treated with the NF-κB inhibitors BMS345541 or TPCA-1 (Cayman Chemical), the culture medium was changed to a medium containing the desired concentration of the inhibitor 1 h prior to the application of *Salmonella* and maintained throughout the remainder of the infection. For infections with Z-VAD-FMK and GSK872, the culture media were changed to medium containing the desired concentration of the inhibitors 2 h prior to the application of *Salmonella* and maintained throughout the remainder of the infection.

### *Salmonella* intramacrophage quantification

For *Salmonella* intramacrophage survival studies, infected macrophages were lysed in 1% Triton X-100 at designated timepoints post-infection. Lysates were serially diluted and plated on LB agar to determine the number of intracellular colony-forming units (CFU). Percent intramacrophage *Salmonella* survival was determined as the number of CFU at the designated timepoint divided by the number of CFU at 1 hpi.

### Macrophage cytotoxicity assay

For quantification of macrophage cell death, the concentration of lactate dehydrogenase (LDH) in supernatants was measured using the CytoTox96 Non-Radioactive Cytotoxicity Assay (Promega) per manufacturer’s instructions. Percent cytotoxicity was calculated as [(experimental release − spontaneous release)/(maximum release − spontaneous release)] × 100.

### Western blots

Infected macrophages were lysed in 1× RIPA buffer (Cell Signaling Technology) with added 1× Protease and Phosphatase Inhibitor Cocktails (Cell Signaling Technology) at designated timepoints. Total protein was quantified using the BCA Protein assay kit (Pierce). Fifty micrograms of total protein was combined with SDS loading dye and heated at 95°C for 5 min before proteins were separated on a 4%–15% SDS polyacrylamide gel (Bio-Rad). Proteins were wet transferred to an Immobilon-P polyvinylidene fluoride (PVDF) membrane (Millipore-Sigma), then blocked for 1 h with 5% bovine serum albumin (BSA, Research Products International). Antibodies are listed in Table S1. Primary antibody incubations were done at 1:1,000 (β-actin at 1:2,000) in BSA overnight at 4°C. Membranes were washed three times in 1× Tris-buffered saline with 0.05% Tween (TBST), then incubated with secondary horseradish peroxidase-conjugated antibody for 1 h at room temperature in 5% milk. After three more washes, chemiluminescent substrate was applied (Pierce ECL western blotting substrate, Thermo Scientific). Chemiluminescence was detected either by exposure to CL-XPosure film (Thermo Scientific) and developed using an AFP Imageworks MM90 or AzureSpot Pro (Azure Biosystems). Chemiluminescence signal for phospho-NF-κB p65 or phospho-STAT1 was normalized to total NF-κB p65 or total STAT1, respectively, or to β-actin.

### TUNEL staining

Adherent infected macrophages were removed from infection wells by adding ice-cold PBS with 0.5 M EDTA for 5 min, then pipetting to loosen macrophages. Cells were fixed using IC Fixation Buffer (Invitrogen) for 20 min at room temperature. After washing, macrophages were permeabilized in 70% ethanol and kept at −20°C until TUNEL staining. TUNEL staining was performed per the manufacturer’s protocol for the Apo-BrdU TUNEL Kit (Phoenix Biosystems). Stained cells were analyzed on an LSRII cytometer (Becton Dickinson). Data were acquired with DIVA software (BD Biosciences) and analyzed using FlowJo software (TreeStar).

### Measurement of NF-κB activity

THP-1 cells transfected with an NF-κB reporter system (THP-1 Blue) were obtained from Invivogen (San Diego, CA) and cultured as above in RPMI 1640 with 10% heat-inactivated FBS, sodium pyruvate (Corning Inc.), non-essential amino acids (Gibco), 50 U mL^−1^ of penicillin, 50 µg mL^−1^ of streptomycin, and 100 µg mL^−1^ of normocin at 37°C in 5% CO2; cells were cultured with added 10 µg mL^−1^ of blasticidin every other passage. THP-1 Blue cells were infected as above. Upon NF-κB activation, secreted embryonic alkaline phosphatase (SEAP) was measured in cell supernatants by the addition of Quanti-Blue (Invivogen) per the manufacturer’s instructions. The assay is read spectrophotometrically on an OptiMax Tunable Microplate reader (Molecular Devices, Sunnyvale, CA) at OD_622_.

### Real-time quantitative reverse transcription PCR (qRT-PCR)

Cellular RNA was collected from THP-1 macrophages infected with *Salmonella* for 24 h using RNAprotect Cell Reagent (Qiagen). RNA was extracted using RNAeasy kit (Qiagen) and cDNA reverse transcribed from 250 ng RNA using iScript kit (BioRad). Quantification by qPCR was performed using iTaq Universal Probes Supermix (BioRad) and exon-spanning TaqMan FAM-MGB probes (ThermoFisher; CXCL10 Hs00171042, TNF Hs00174128) on a QuantStudio 3 thermocycler (ThermoFisher). All qPCRs were run in technical triplicate. Mean comparative threshold cycle (CT) value for each transcript was adjusted for input variation by subtracting the mean 18 s (RNA18S5; ThermoFisher Hs03928990) housekeeping control CT from the target gene’s CT to generate a ΔCT. The ΔΔCT for each knockdown was calculated by subtracting target gene ΔCT in uninfected control cells from target gene ΔCT in infected cells. Knockdown fold change was then calculated as 2-ΔΔCT.

### PBMC-derived macrophage cell harvest

Leukocyte reduction filters (Bloodworks NW, Seattle, WA) were backflushed with 100 mL of sterile PBS with 5 mM EDTA. Eluate was overlaid on Ficoll-Paque PLUS (GE Healthcare) and centrifuged at 25°C for 35 min at 2,000 rpm (brake off). Following gradient separation, the monocyte-containing layer was removed and washed twice in sterile PBS. Cells were filtered using a sterile 70 µM nylon mesh cell strainer (Fisherbrand) and suspended in RPMI 1640 with 10% human AB serum (Corning Inc.), sodium pyruvate (Corning Inc.), non-essential amino acids (Gibco), 50 U mL^−1^ of penicillin, 50 µg mL^−1^ of streptomycin, and 10 ng mL^−1^ of granulocyte-macrophage colony-stimulating factor (GM-CSF) (PeproTech). Cells were incubated in untreated tissue culture flasks for 48 h; adherent cells were then scraped and seeded into tissue culture wells and allowed to adhere for 5 days. The medium was exchanged for GM-CSF-free, antibiotic-free medium 24 h prior to infection; PBMC-derived macrophages were infected as with THP-1-macrophages above.

### Cytokine ELISAs

Culture medium supernatants were collected from infected THP-1- and PBMC-derived macrophages 24 hpi and analyzed for IL-12 using the Human IL-12/IL-23 p40 DuoSet and Human IL-12 p70 DuoSet from R&D Systems, performed following the manufacturer’s protocols.

### Intracellular cytokine staining

Infected PBMC-derived macrophages were treated with Brefeldin A (Thermo Scientific) to a final concentration of 3.0 µg mL^−1^ in infection wells for 2 h prior to fixation and staining. Cells were washed once with PBS and scraped into ice-cold PBS with 0.5 M EDTA in round-bottom polypropylene tubes. Cells were washed once by centrifugation at 600 × *g* for 5 min at 25°C and resuspended at approximately 1 × 10^6^ cells mL^−1^ in PBS. Cells were stained with fixable viability dye (Thermo Scientific) at 1:1,000 for 30 min on ice, then washed with Flow Cytometry Staining Buffer (Thermo Scientific). Fc receptor binding inhibitor was added for 20 min on ice, then surface antigens were stained for 30 min on ice in the dark. Cells were washed with Flow Cytometry Staining Buffer followed by fixation with IC Fixation Buffer (Thermo Scientific) for 20 min at room temperature. Cells were washed twice with Permeabilization Buffer (Thermo Scientific) and stained for intracellular hIL-12p70 for 30 min at room temperature in the dark. After washing, cells were resuspended in Flow Cytometry Staining Buffer and analyzed on an LSRII cytometer (Becton Dickinson). Data were acquired with DIVA software (BD Biosciences) and analyzed using FlowJo software (TreeStar).

### Humanized mouse infections

Mouse experiments in this study were approved by the University of Washington Institutional Animal Care and Use Committee (IACUC) and performed as described in protocol 3373-01. NOD-*Prkdc^scid^IL2rg^tm1Wjl^* (NSG) mice were purchased from The Jackson Laboratory (Bar Harbor, ME) and engrafted with human CD34+ hematopoietic stem cells derived from the umbilical cord blood (83, 84). Umbilical cord blood was obtained from donors that were consented under an approved IRB protocol at the UMass Memorial Medical Center, Department of General Obstetrics and Gynecology (Worcester, MA), and all samples used for engraftment were de-identified. Mice were maintained under ABSL-2 containment at the University of Washington Animal Care and Research Facility on a 14-h light cycle and housed up to five animals per cage in Allentown cages with micro-isolator tops. Mice were checked daily during infection studies, and veterinary care was provided 7 days a week.

CD34+Hu NSG mice were infected with a total of ~6 × 10^4^ CFU of wild-type *S*. Typhi, ~1 × 10^3^ CFU of wild-type *S*. Typhimurium, or ~1 × 10^3^ CFU of *S*. Typhimurium Δ*ssrB* (85). Infection inocula were chosen to obtain similar infection lengths between strains. Infected mice were closely monitored for signs of illness and humanely euthanized when moribund or 7 days post-infection, whichever came first. Intracardiac blood was harvested and centrifuged in serum separator tubes (BD Biosciences) for 5 min at 15,000 rpm before storage at −80°C. Serum cytokines were measured using a multiplex bead array assayed by Luminex 200. Livers and spleens were aseptically harvested and homogenized in PBS using a Power Gen 125 tissue homogenizer (Fisher Scientific), then serially diluted and plated on LB agar + “aromix” for CFU counts.

### Statistical analysis

Statistical method and sample size for experiments are indicated in the corresponding figure legends. Statistical analysis of macrophage and mouse infections was performed using Prism v. 8.1.2 software (GraphPad). Statistical significance was defined as *P* ≤ 0.05. Error bars on figures show standard deviation.

## Data Availability

All data are available in the paper or the supplemental material. CD34+Hu NSG mice are available from The Jackson Laboratory. Correspondence and material requests should be addressed to the corresponding author.

## SUPPLEMENTAL MATERIAL

Supplemental Material mbio.00454-24-s0001.pdf Supplemental figures and tables.

ASM does not own the copyrights to Supplemental Material that may be linked to, or accessed through, an article. The authors have granted ASM a non-exclusive, world-wide license to publish the Supplemental Material files. Please contact the corresponding author directly for reuse.
