# Supplementary material for: Nuclear factor kappa B-dependent persistence of Salmonella Typhi and Paratyphi in human macrophages
Source: mBio. 2024 Mar 18;15(4):e00454-24. doi: 10.1128/mbio.00454-24 (PMC11005419; doi:10.1128/mbio.00454-24)
Supplement: Supplemental Material — Supplemental figures and tables. [file mbio.00454-24-s0001.pdf]

SUPPLEMENTAL FIGURES AND TABLES

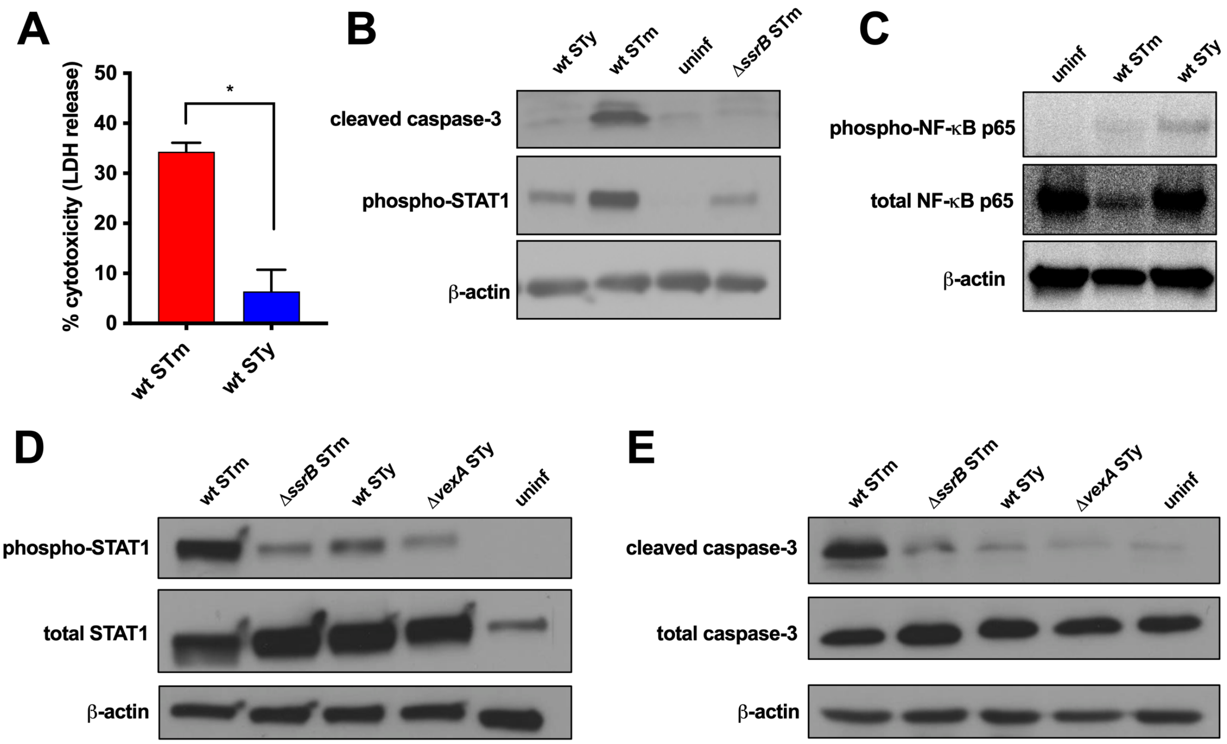

Supplementary Figure 1. Differential cytotoxicity of *S.* Typhi and *S.* Typhimurium for human macrophages. (**A-B**) Observations from infections in THP-derived macrophages were confirmed in macrophages derived from PBMCs. Macrophages were infected with opsonized stationary-phase *Salmonella* at an MOI of 10:1. *S.* Typhi Ty2 = STy, *S.* Typhimurium 14028s = STm. (**A**) Macrophage cytotoxicity was measured as the amount of LDH released in supernatants 24 hpi with wild-type or isogenic mutant *Salmonella* strains, n=3. Bar graphs represent the means of three separate experiments with error bars representing standard deviations. Statistical significance was determined by paired two-tailed Student's t test; * p≤0.05. (**B**) Fifty µg of total protein from cells infected with *Salmonella* for 24 h were subjected to western blot analysis for cleaved caspase-3 or phosphorylated STAT1, with measurement of β-actin included as a loading

control. Results from one representative experiment of three are shown. (**C-E**) Fifty μg of total protein from THP-1 cells infected with *Salmonella* for 24 h were subjected to western blot analysis for phospho-NF-κBp65/total NF-κBp65 (**C**), cleaved caspase-3/procaspase-3 (**D**), phospho-STAT1/total STAT1 (**E**), with measurement of β-actin included as a loading control. Results from one representative experiment of three are shown.

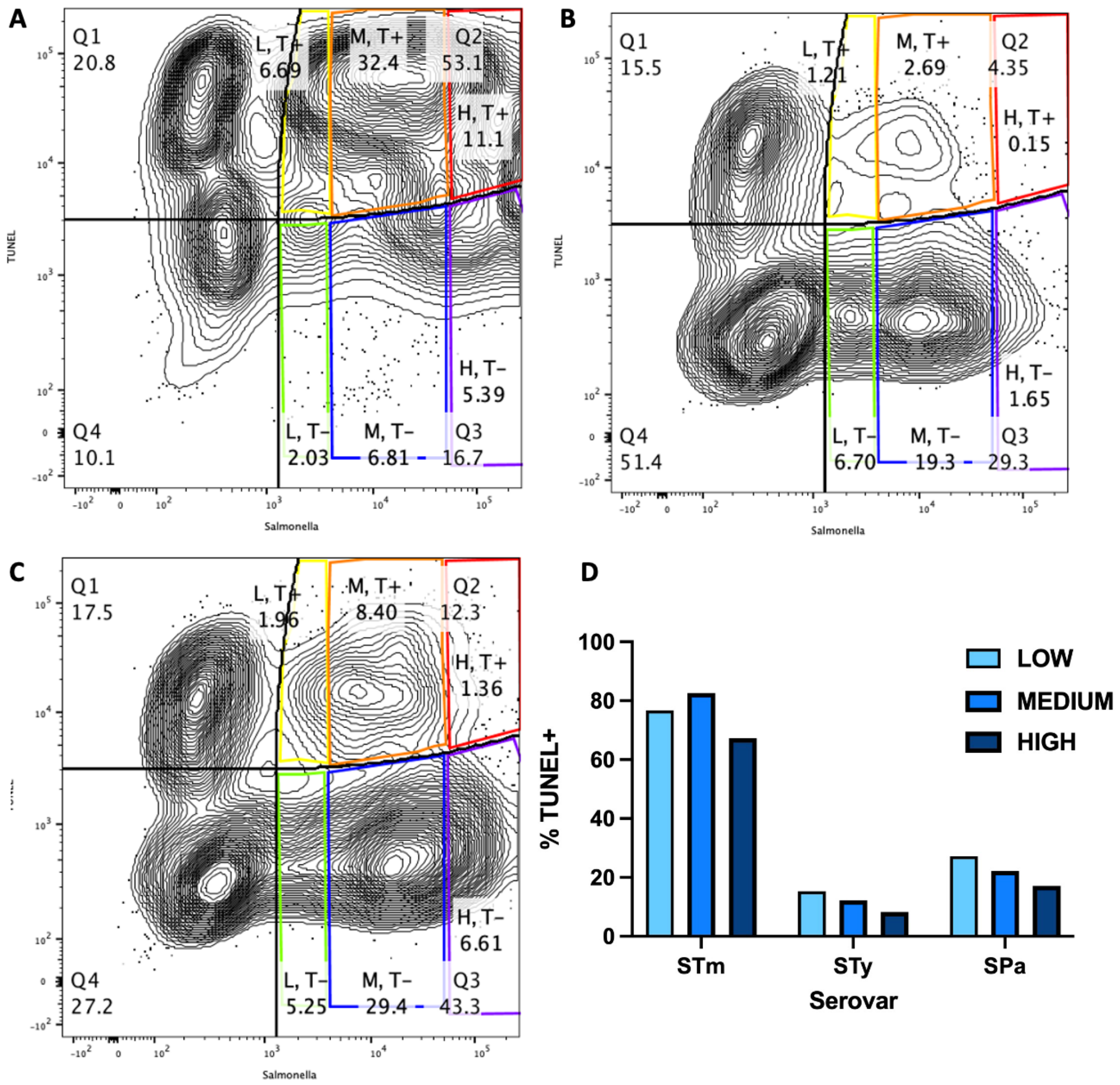

Supplementary Figure 2. Bacterial load and macrophage apoptosis. Macrophages infected with wild-type YPet-expressing (**A**) *S.* Typhimurium, (**B**) *S.* Typhi, or (**C**) *S.* Paratyphi A were stratified according to low (L), medium (M), or high (H) bacterial load based on fluorescence signal intensity, and apoptosis was measured by TUNEL (T) assay. (**D**) Macrophage apoptosis was dependent on serovar but not on bacterial load. The percent of total events for each grouping is shown.

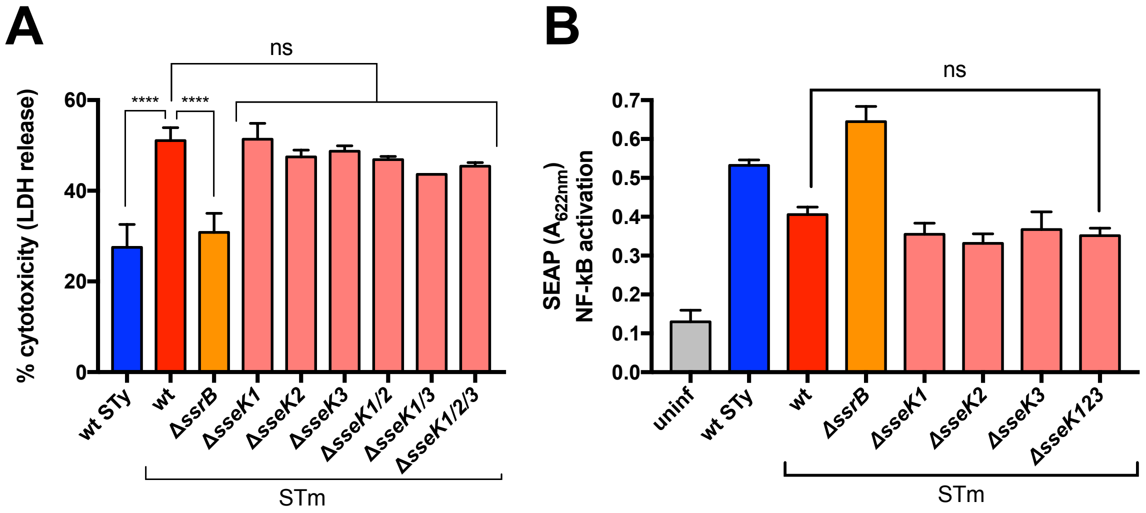

Supplementary Figure 3. The redundant SPI2 Effectors SseK1, SseK2, and SseK3 are not responsible for cell death or NF-kB activation. THP-1 cells were differentiated with PMA and infected with opsonized stationary-phase *Salmonella* at an MOI of 10:1. *S.* Typhi Ty2 = STy, *S.* Typhimurium 14028s = STm. (**A**) Macrophage cytotoxicity was measured as the amount of LDH released in supernatants 24 hpi with wild-type or isogenic mutant *Salmonella* strains, n=3. (**B**) THP-1 NF-kB Blue reporter cells were infected with *Salmonella* and NF-kB activation measured 24 hpi using the colorimetric Quanti-Blue assay. Statistical significance was determined by one-way ANOVA with Sidak's multiple comparison test; * p≤0.0332; ** p≤0.0021; *** p≤0.002; **** p≤0.0001.

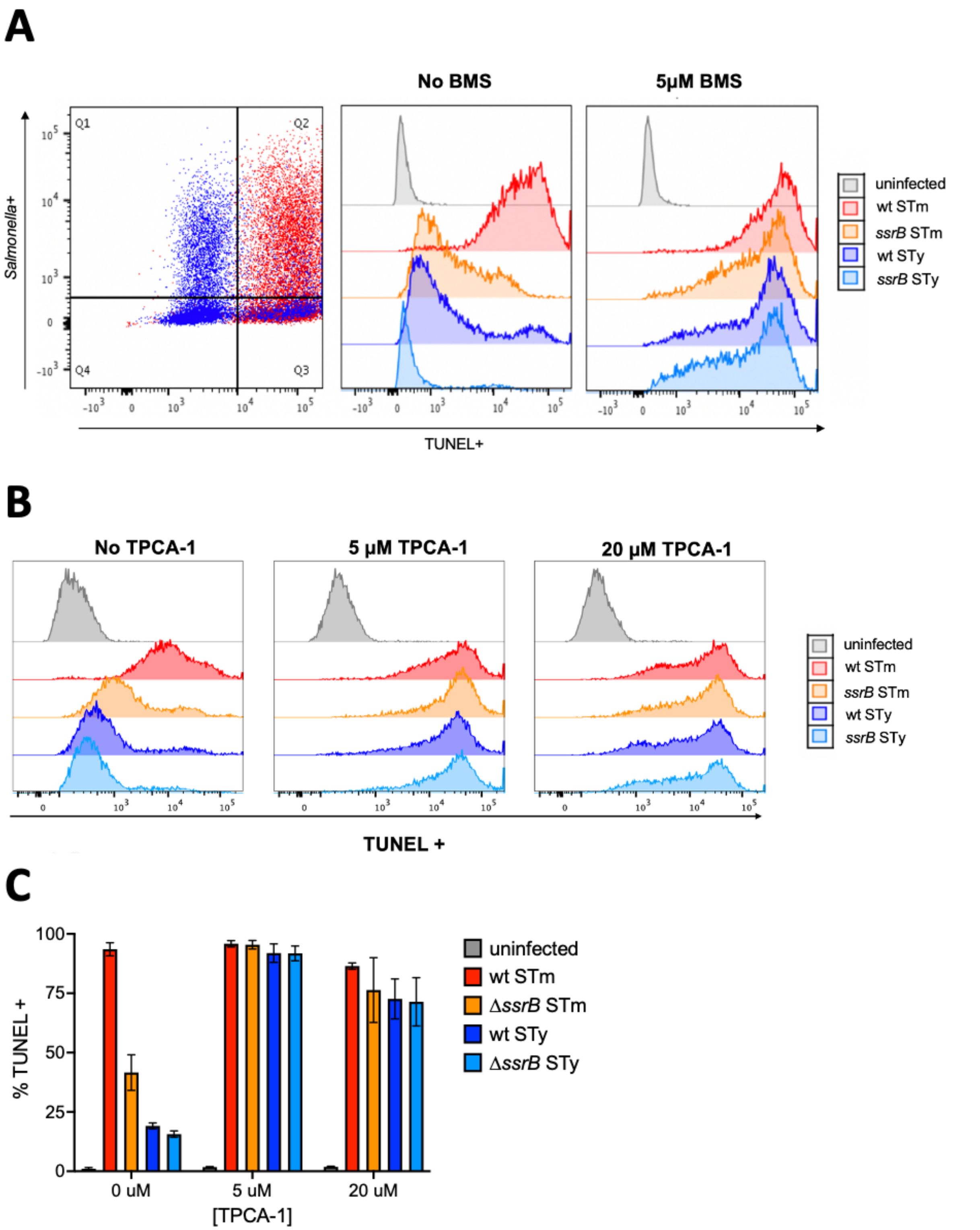

Supplementary Figure 4. Pharmacologic inhibition of NF-κB induces apoptosis of *Salmonella*- infected macrophages. THP-1 cells were differentiated with PMA and

infected with opsonized stationary-phase *Salmonella* at an MOI of 10:1. Macrophages were treated with the indicated dose of the NF-kB inhibitors BMS345541 or TPCA-1 1 h prior to infection and treatment maintained throughout the infection. Macrophages were stained for TUNEL 24 h after *Salmonella* infection. *S.* Typhimurium 14028s = STm, *S.* Typhi Ty2 = STy, *S.* Paratyphi A ATCC9150 = SPa. **(A)** Representative raw data are shown from experiment depicted in Fig. 5D using BMS345541. (**B-C**) Experiment using inhibitor TPCA-1.

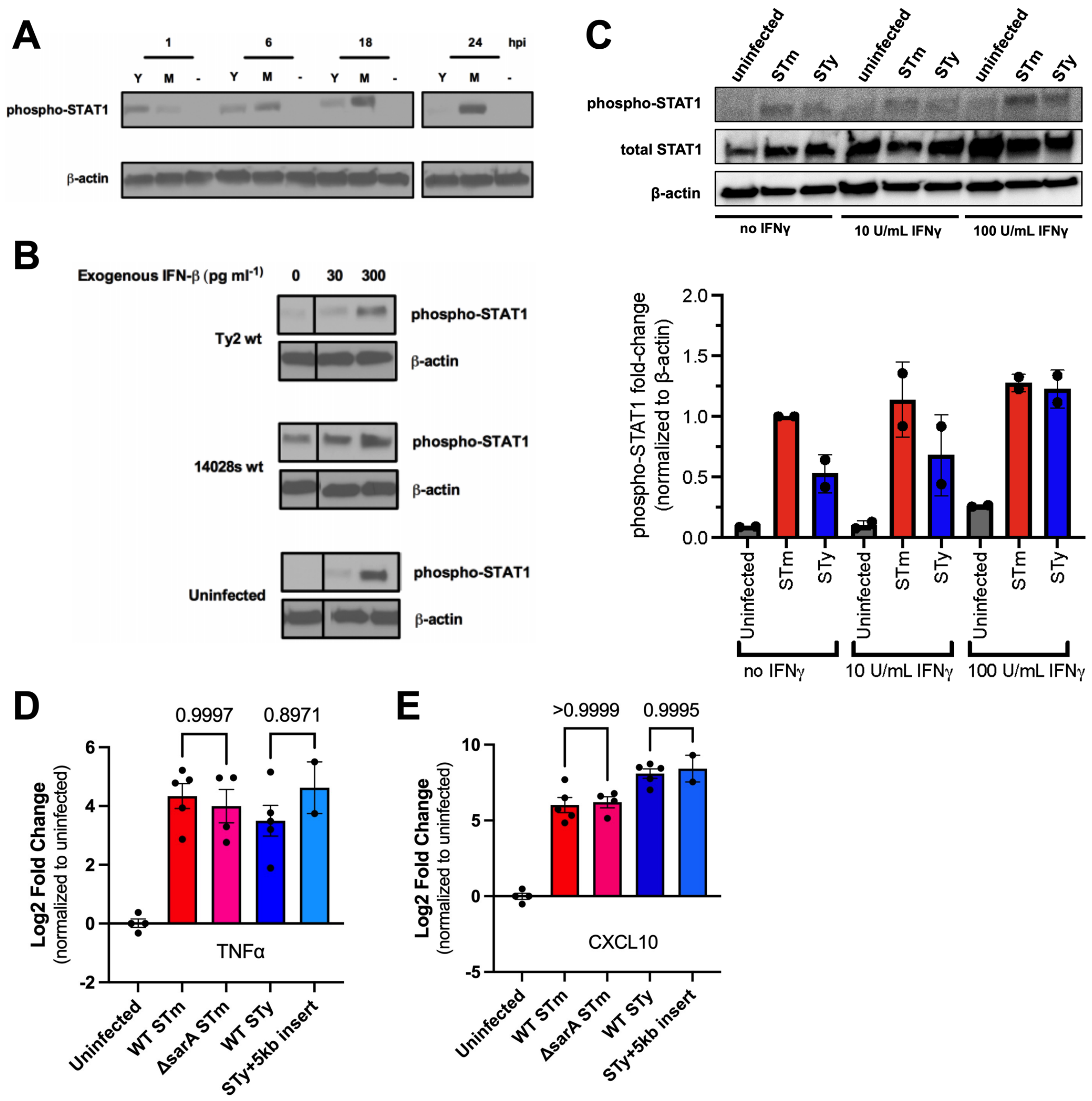

Supplementary Figure 5. *S.* Typhi promotes STAT1 dephosphorylation. THP-1 cells were differentiated with PMA and infected with opsonized stationary-phase *Salmonella* at an MOI of 15:1. Wild-type *S.* Typhi Ty2 = Y / Ty2 / STy; wild-type *S.* Typhimurium 14028s = M / 14028s / STm; uninfected = –. **(A)** Fifty μg of total protein from THP-1 cells infected with *Salmonella* for the indicated amount of time were subjected to western blot analysis for phosphorylated STAT1. Early after infection (1 and 6 hpi), phospho-STAT1 levels were

similarly increased in both *S.* Typhi- and *S.* Typhimurium-infected cells, while at later timepoints *S.* Typhi-infected cells contained reduced levels of phospho-STAT1, suggesting that *S.* Typhi may promote STAT1 dephosphorylation. (**B-C**) Recombinant human IFNβ or IFNγ was administered to THP-1 macrophages 24 h prior to infection to stimulate STAT1 phosphorylation. Fifty *μ*g of total protein from THP-1 cells 24 hpi were subjected to western blot analysis for phosphorylated STAT1. Measurement of β-actin was included as a loading control. Treatment of uninfected or *Salmonella*-infected cells with exogenous IFNβ induced STAT1 phosphorylation, but *S.* Typhi-infected cells exhibited lower levels of STAT1 phosphorylation in comparison to *S.* Typhimurium-infected cells. A high dose of IFNγ induced STAT1 phosphorylation in both *S.* Typhi- and *S.* Typhimurium-infected cells. (**D-E**) Expression of STAT1-stimulated TNFα and CXCL10 genes was measured by qRT-PCR of cellular RNA collected from THP-1 macrophages infected with *Salmonella* for 24 h. The 5kb insert carries *gogB, sarA* and *pagK2*. Mean log2 fold-change ±SEM is reported. Statistical significance was determined using a Dunnett's T3 multiple comparisons test after a Brown-Forsythe and Welch ANOVA test.

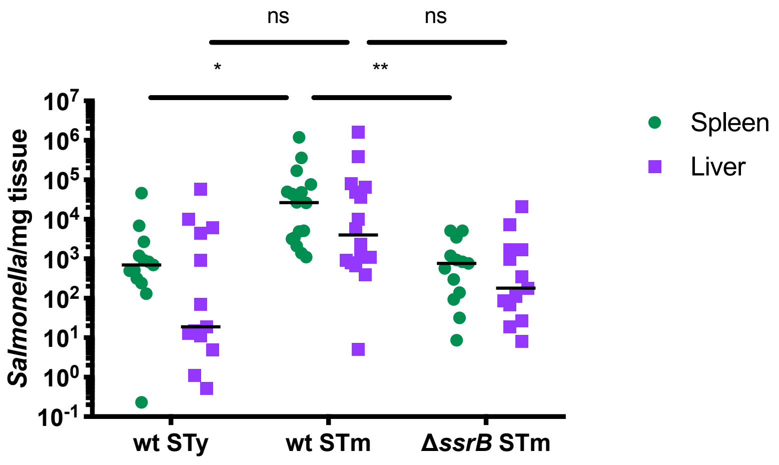

Supplementary Figure 6. Organ burdens of infected humanized mice. CD34+ Hu-NSG mice were infected with wild-type *S.* Typhi, wild-type *S.* Typhimurium, or *ssrB* mutant *S.* Typhimurium, and spleens and livers aseptically removed after mice were humanely euthanized. Organs were serially diluted in PBS and plated on LB agar to determine the number of CFU per mg tissue. Statistical significance was determined by Mann-Whitney test,

* $p \leq 0.05$, ** $p \leq 0.01$.

Supplementary Table 1. Strains, Plasmids, and Reagents

**Bacterial Strains**		
Salmonella enterica serovar Typhimurium 14028s wild-type	S. Miller	JK1324, LAS5, TAS8
S. Typhimurium 14028s *ΔinvA::FRTaphFRT*	1	JK1217
S. Typhimurium 14028s *ΔssrB::FRTaphFRT*	This study	LAS18, TAS9
S. Typhimurium 14028s *ΔssaGH::FRTaphFRT*	This study	TAS210
S. Typhimurium 14028s *ΔavrA::FRTaphFRT*	This study	TAS12
S. Typhimurium 14028s *ΔcigR::FRTaphFRT*	This study	TAS20
S. Typhimurium 14028s *ΔgogA::tetRA*	This study	FEG43
S. Typhimurium 14028s *ΔgogB::FRTaphFRT*	This study	LAS86
S. Typhimurium 14028s *ΔgtgA::FRTaphFRT*	This study	FEG45
S. Typhimurium 14028s *ΔgtgE::FRTaphFRT*	This study	TAS22
S. Typhimurium 14028s *ΔsarA::FRTaphFRT*	This study	TAS199
S. Typhimurium 14028s *ΔslrP::FRTaphFRT*	This study	LAS82
S. Typhimurium 14028s *ΔsopD2::FRTaphFRT*	This study	LAS83
S. Typhimurium 14028s *ΔspvB::FRTaphFRT*	This study	LAS85
S. Typhimurium 14028s *ΔspvC::FRTaphFRT*	This study	LAS81
S. Typhimurium 14028s *ΔspvD::FRTaphFRT*	This study	LAS84
S. Typhimurium 14028s *ΔspvR::FRTaphFRT*	This study	LAS16
S. Typhimurium 14028s *ΔsrfJ::FRTaphFRT*	This study	LAS211
S. Typhimurium 14028s *ΔsseI::FRTaphFRT*	This study	LAS90
S. Typhimurium 14028s *ΔsseJ::FRTaphFRT*	This study	LAS88
S. Typhimurium 14028s *ΔsseK1::FRTaphFRT*	This study	TAS10
S. Typhimurium 14028s *ΔsseK2::FRTaphFRT*	This study	LAS87
S. Typhimurium 14028s *ΔsseK3::FRTaphFRT*	This study	LAS80
S. Typhimurium 14028s *ΔsspH1::FRTaphFRT*	This study	LAS89
S. Typhimurium 14028s *ΔsteB::FRTaphFRT*	This study	TAS18
S. Typhimurium 14028s *ΔsseK1::FRT ΔsseK2::FRTaphFRT ΔsseK1::FRTcatFRT*	This study	TAS255
S. Typhimurium 14028s ΔgogA::tetRA ΔgtgA::FRTaphFRT	This study	FEG46
S. Typhimurium 14028s *ΔsarA::FRTaphFRT* / pTS11 *bla*	This study	TAS214
S. Typhimurium 14028s wild-type / pBC19-Ypet *aph*	This study	JK1606
S. Typhimurium 14028s *ΔssrB::FRTaphFRT* / pBC19-Ypet *aph*	This study	JK1619
S. Typhimurium 14028s wild-type / p67GFP3.1	2	DCK22
S. Typhimurium 14028s *ΔSTM2585* / p67GFP3.1	3	DCK444
Salmonella enterica serovar Typhi Ty2 wild-type JSG624	J. Gunn	TY2
S. Typhi Ty2 Nal^R (mouse infections)	This study	TY196
S. Typhi Ty2 *ΔssrB::FRTaphFRT*	4	TY144
S. Typhi Ty2 wild-type / pBC19-Ypet *aph*	This study	TY399
S. Typhi Ty2 *ΔvexA::FRTaphFRT*	4	TY76
S. Typhi Ty2 *ΔvexA::FRT*	This study	TY181
S. Typhi Ty2 *ΔvexA::FRT* / pJK724 *bla*	This study	TY478
S. Typhi Ty2 *ΔvexA::FRT* / pJK761 *bla*	This study	TY479
S. Typhi Ty2 *ΔvexA::FRT* / pJK762 *bla*	This study	TY480
S. Typhi Ty2 *ΔvexA::FRT* / pJK763 *bla*	This study	TY481
S. Typhi Ty2 *ΔvexA::FRT* / pJK764 *bla*	This study	TY482
S. Typhi Ty2 *ΔvexA::FRT* / pJK765 *bla*	This study	TY483
S. Typhi Ty2 *ΔvexA::FRT* / pJK766 *bla*	This study	TY484

S. Typhi Ty2 *ΔvexA*::*FRT* / pJK767 *bla*		This study	TY485
S. Typhi Ty2 *ΔvexA*::*FRT* / pJK768 *bla*		This study	TY486
S. Typhi Ty2 *ΔvexA*::*FRT* / pJK769 *bla*		This study	TY487
S. Typhi Ty2 wild-type / p67GFP3.1		5	DCK33
S. Typhi Ty2 wild-type / p67GFP3.1 and pWSK129+5kbpIL10		This study	DCK424
Salmonella enterica serovar Paratyphi A ATCC 9150 wild-type		ATCC	TY363
S. Paratyphi A ATCC 9150 *ΔssrB::FRTaphFRT*		This study	TY377
S. Paratyphi A ATCC 9150 wild-type / pBC20-Ypet *aph*		This study	TY407
S. Paratyphi A ATCC 9150 / pJK724 *bla*		This study	TY591
S. Paratyphi A ATCC 9150 / pJK762 *bla*		This study	TY593
S. Paratyphi A ATCC 9150 / pJK764 *bla*		This study	TY595
**Plasmids**			
araC-P$_{araB}$-$\gamma\beta$*exo* oriR101 repA101ts *bla*		6	pKD46
FRTaphFRT PS1 PS2 oriR *bla*		6	pKD4
FRTcatFRT PS1 PS2 oriR *bla*		6	pKD3
FRTaphFRT PS1 PS4 oriR *bla*		6	pKD13
cI857 lPr *flp* PSC101 oriTS *bla cat*		6	pCP20
Medium copy cloning vector ori pBR322 *bla*		This study	pJK392
pJK392::STM*sarA bla*		This study	pTS11
P*ybaJ*-Ypet 10% ori pSC101 *aph*		D. Bumann	pBC19
P*ybaJ*-Ypet 3% ori pSC101 *aph*		D. Bumann	pBC20
Low-to-medium copy cloning vector *par* RK2 oriV *trfA bla*		7	pRB3-273C
Cloning vector Ptrc ori ColE1 *bla*		8	pTrc99a
pRB3-273C-pTRC *bla*		This study	pJK724
pRB3-273C-pTRC-STM$_{avrA}$ *bla*		This study	pJK761
pRB3-273C-pTRC-STM$_{gogA}$ *bla*		This study	pJK762
pRB3-273C-pTRC-STM$_{gogB}$ *bla*		This study	pJK763
pRB3-273C-pTRC-STM$_{gtgA}$ *bla*		This study	pJK764
pRB3-273C-pTRC-STM$_{sspH1}$ *bla*		This study	pJK765
pRB3-273C-pTRC-STM$_{sseK1}$ *bla*		This study	pJK766
pRB3-273C-pTRC-STM$_{sseK2}$ *bla*		This study	pJK767
pRB3-273C-pTRC-STM$_{sseK3}$ *bla*		This study	pJK768
pRB3-273C-pTRC-STM$_{spvD}$ *bla*		This study	pJK769
pRB3-273C-pTRC *bla*		This study	pJK770
RSF1010 derivative expressing inducible *gfp*		9	p67GFP3.1
Low copy cloning vector ori PSC101 *aph*		10	pWSK129
**Cell Lines**			
THP-1 monocyte	ATCC	ATCC TIB-202	RRID:CVCL_0006
THP-1 Blue NF-κB Reporter	Invivogen	Cat# thp-nfkb	
**Mouse Lines**			
NOD-*scid* $IL2r\gamma^{null}$ mice	Dale Greiner, University of Massachusetts	NSG	
Humanized-NOD-*scid* $IL2r\gamma^{null}$ mice	Dale Greiner, University of Massachusetts	CD34+ Hu-NSG	
**Antibodies**			
Rabbit monoclonal anti-phosphoSTAT1 (Y701, clone D47A)	Cell Signaling Technologies	Cat#7649	RRID:AB_10950970

Rabbit polyclonal anti-STAT1	Cell Signaling Technologies	Cat#9172	RRID: AB_2198300
Rabbit monoclonal anti-cleaved Caspase-3 (Asp175, clone 5A1E)	Cell Signaling Technologies	Cat#9664	RRID:AB_2070042
Rabbit monoclonal anti-phospho-NF-κB p65 (Ser536, clone 93H1)	Cell Signaling Technologies	Cat#3033	RRID:AB_331284
Rabbit monoclonal anti- NF-κB p65 (clone D14E12)	Cell Signaling Technologies	Cat#8242	RRID: AB_10859369
Rabbit monoclonal anti-beta-Actin	Cell Signaling Technologies	Cat#4967	RRID:AB_330288
Goat anti-Rabbit IgG, HRP-linked	Cell Signaling Technologies	Cat#7074	RRID:AB_2099233
Mouse monoclonal anti-BrdU Alexa Fluor647-conjugated (clone 3D4)	BioLegend	Cat#364108	RRID:AB_2566452
Mouse monoclonal anti-BrdU (clone PRB-1) FITC-conjugated	Phoenix Flow Systems	Cat#ABFM-18	
Mouse monoclonal anti-CD11b PECy7-conjugated (clone ICRF44)	Invitrogen	Cat#25-0118-42	RRID:AB_1582272
Mouse monoclonal anti-IL-12p35 PE-conjugated (clone 27537)	Invitrogen	Cat#MA5-23559	RRID:AB_2609031
Mouse IgG1 kappa isotype control PE-conjugated	Invitrogen	Cat#12-4714-41	RRID:AB_1944424
**Chemicals, Peptides, and Recombinant Protiens**			
RPMI 1640, 1x with L-glutamine and 25mM HEPES	Corning	Cat#10-041-CV	
Sodium Pyruvate 100mM solution	Corning	Cat#25-000-CI	
MEM Non-Essential Amino Acids 100X	Gibco	Cat#11140-050	
Penicillin Streptomycin	Corning	Cat#30-001-CI	
Fetal Bovine Serum, heat-inactivated (USA sourced)	Millipore-Sigma	F4135	
Phorbal 12-myristate 13-acetate	Millipore-Sigma	P1585	
Human pooled serum	MP Biomedicals LLC	Cat#2930149	
Phosphate-buffered saline	Corning	Cat#21-040-CV	
Triton X-100	Fisher BioReagents	Cat#BP151-100	
LB Broth, Miller	Fisher BioReagents	Cat#BP1426	
LB Agar, Miller	Fisher BioReagents	Cat#BP1425	
L-Phenylalanine	Millipore-Sigma	Cat#P2126	
L_Tryptophan	Millipore-Sigma	Cat#T0254	
2,3-Dihydroxybenzoic acid	Millipore-Sigma	Cat#126209	
4-Aminobenzoic acid	Millipore-Sigma	Cat#A9878	
2,6-Diaminopimelic acid	Millipore-Sigma	Cat#33240	
Ampicillin, sodium salt	Research Products International	Cat#A40040	
Carbenicillin disodium salt	Research Products International	Cat#C46000	
Kanamycin sulfate	VWR	Cat#0408	
Gentamicin sulfate	Research Products International	Cat#G3800	
Normocin	Invivogen	Cat#ant-nr-1	

Blasticidin	Invivogen	Cat#ant-bl-05	
Ficoll-Paque PLUS	GE Healthcare	Cat#17-1440-02	
hGM-CSF	PeproTech		
Human AB serum	Corning	Cat#35-060-CI	
Lowfat powdered milk	Saco Foods Inc.		
Bovine serum albumin	MP Biomedicals LLC	Cat#30075	
Brefeldin A	Invitrogen	Cat#00-4506-51	
Fixable viability dye eFluor450	Invitrogen	Cat#65-0863-14	
IC Fixation Buffer	Invitrogen	Cat#00-8222-49	
Permeabilization Buffer 10x	Invitrogen	Cat#00-8333-56	
EDTA 0.5M	Corning	Cat#14-034-CI	
Quanti-Blue solution	Invivogen	Cat#rep-qbs	
Pierce ECL western blotting substrate	ThermoFisher Scientific	Cat#32209	
RIPA buffer	Cell Signaling Technologies		
Phosphatase inhibitor cocktail	Cell Signaling Technologies	Cat#5870	
Protease inhibitor cocktail	Cell Signaling Technologies	Cat#5871	
BMS345541	Cayman Chemical	Item#16667	
TPCA-1	Cayman Chemical	Item#15115	
GSK872	Cayman Chemical	Item#23300	
Recombinant human IFNβ	PeproTech	Cat#300-02BC	
Recombinant human IFNγ	PeproTech	Cat#300-02	
Z-VAD-FMK	BD Biosciences	Cat#550377	

Supplementary Table 2. Primers

Primer	Sequence 5'-3'	Purpose
TYP45	ATCATCATATTACTAACGACATTTTTCCTGCTTTCGGGATGTGTAGGCTGGAGCTGCTTC	Deletion of *vexA* in STy
TYP46	TTAGTGCCGCGGGTCAAAAAGCTATCGAATGCGGCTTTCACATATGAATATCCTCCTTAG	Deletion of *vexA* in STy
TYP13	TATAAGATCTTATTAGTAGACGATCATGAAATCATCATTAGTGTAGGCTGGAGCTGCTTC	Deletion of *ssrB* in STy
TYP14	ATTAACCTCATTCTTCGGGCGCAGTTAAGTAACTCTGTCACATATGAATATCCTCCTTAG	Deletion of *ssrB* in STy
JKP696	CGCGAGGGCAGCAAAATGAAAGAATATAAGATCTTATTAGGTGTAGGCTGGAGCTGCTTC	Deletion of *ssrB* in STm
JKP697	AGTTAAGTAACTCTGTCACTTTATGAACCTGTAGCTTTCTC	Deletion of *ssrB* in STm
TSP481	GTCAAAGTAATACTCAAACCATCGCACCTACGCTCAGTCCGTGTAGGCTGGCGCTGCTTC	Deletion of *sseK2* in STm
TSP482	TTACCTCCAAGAACTGGCAGTTAAACTGCTTGTGTTCATACATATGAATATCCTCCTTAG	Deletion of *sseK2* in STm
TSP485	ATGTTTTCTCGAGTCAGAGGTTTTCTTTCATGCCAGAACTGTGTAGGCTGGAGCTGCTTC	Deletion of *sseK3* in STm

TSP486	TTATCTCCAGGAGCTGATAGTCAAACTGCTGGTATCCATACATATGAATATCCTCCTTAG	Deletion of *sseK3* in STm
TSP1	ATGATCCCACCATTAAATAGATATGTTCCCGCGCTTTCAATGTGTAGGCTGGAGCTGCT	Deletion of *sseK1* in STm
TSP2	CTACTGCACATGCCTCGCCCATGAACTTTGCGTAAACTGACATATGAATATCCTCCTTAG	Deletion of *sseK1* in STm
TSP4	TTAGCATAACGGCATTGTTATCGAATCGCTCATAAAGCGTTGTGTAGGCTGGAGCTGCT	Deletion of *avrA* in STm
TSP5	ATGATATTTTCGGTGCAGGAGCTATCATGTGGAGGGAAAACATATGAATATCCTCCTTA	Deletion of *avrA* in STm
TSP13	ATGCCTATTTCGATTTGTAAACATGGTGCTCCTTTTGTTGTGTGTAGGCTGGAGCTGCT	Deletion of *steB* in STm
TSP14	TTATCTGACATTACCATTTGAGTGACAGGTTAGCAGATGTCATATGAATATCCTCCTTA	Deletion of *steB* in STm
TSP16	TTAATCAAATACGCCATTAATAATCGCCGTGACCACCGCGTGTGTAGGCTGGAGCTGCT	Deletion of *cigR* in STm
TSP17	ATGAATAATCGTCGTGGTTTAACCGCCGTCCTGGCGACGTCATATGAATATCCTCCTTA	Deletion of *cigR* in STm
TSP19	TCATAAAATGGTACACCAGTCTTTCCAGGCGGCGGCGTGTTGTGTAGGCTGGAGCTGCT	Deletion of *gtgE* in STm
TSP20	ATGTTAAGACACATTCAAAATAGTTTAGGCAGCGTTTACACATATGAATATCCTCCTTA	Deletion of *gtgE* in STm
slrPkop1	ATGTTTAATATTACTAATATACAATCTACGGCAAGGCATCTGTGTAGGCTGGACCTGCTT	Deletion of *slrP* in STm
slrPkop2	CTATCGCCAGTAGGCGCTCATGAGCGAGCTCACCTCTTTTCATATGAATATCCTCCTTAG	Deletion of *slrP* in STm
sopD2kop1	ATGCCAGTTACGTTAAGTTTTGGTAATCGTCATAACTATGTGTGTAGGCTGGAGCTGCTT	Deletion of *sopD2* in STm
sopD2kop2	TTATATAAGCATATTGCGACAACTCGACTTTTCACTTATACATATGAATATCCTCCTTAG	Deletion of *sopD2* in STm
sseJkop1	ATGCCATTGAGTGTTGGACAGGGTTATTTCACATCATCTATGTGTAGGCTGGAGCTGCTT	Deletion of *sseJ* in STm
sseJkop2	TTATTCAGTGGAATAATGATGAGCTATAAAACTTTCTAACCATATGAATATCCTCCTTAG	Deletion of *sseJ* in STm
sseIkop1	ATGCCCTTTCATATTGGAAGCGGATGTCTTCCCGCCATCATGTGTAGGCTGGAGCTGCTT	Deletion of *sseI* in STm
sseIkop2	TTACATTTTACCTATTAAGGAATATTTTTGCTTTTTAAAGCATATGAATATCCTCCTTAG	Deletion of *sseI* in STm
sspH1kop1	ATGTTTAATATCCGCAATACACAACCTTCTGTAAGTATGCTGTGTAGGCTGGAGCTGCTT	Deletion of *sspH1* in STm
sspH1kop2	TCAGTTAAGACGCCACCGGGCTGTCAGATAGCTACCCAGCCATATGAATATCCTCCTTAG	Deletion of *sspH1* in STm
gogBkop1	TTGACATATAGATTGAAAAAGCGCATGAAAATAGGATTCCTGTGTAGGCTGGAGCTGCTT	Deletion of *gogB* in STm
gogBkop2	TCAACGATTTCTATTTTTAGGCTTATATTTATCCCAACCACATATGAATATCCTCCTTAG	Deletion of *gogB* in STm
sseK2kop1	ATGGCACGTTTTAATGCCGCTTTTACAAGGATTAAAATAATGTGTAGGCTGGAGCTGCTT	Deletion of *sseK2* in STm

sseK2kop2	TTACCTCCAAGAACTGGCAGTTAAACTGCTTGTGTTCATACATATGAATATCCTC CTTAG	Deletion of *sseK2* in STm
spvBkop1	CTATGAGTTGAGTACCCTCATGTTTATTATTCTTTTTATCTGTGTAGGCTGGAGC TGCTT	Deletion of *spvB* in STm
spvBkop2	ATGTTGATACTAAATGGTTTTTCATCTGCCACTTTAGCGCCATATGAATATCCTC CTTAG	Deletion of *spvB* in STm
spvCkop1	TTACTCTGTCATCAAACGATAAAACGGTTCCTCACGTAAATGTGTAGGCTGGAG CTGCTT	Deletion of *spvC* in STm
spvCkop2	ATGCCCATAAATAGGCCTAATCTAAATCTAAACATCCCTCCATATGAATATCCTC CTTAG	Deletion of *spvC* in STm
spvDkop1	TCAATCGTGTTTTTCATCATAAGCCCTGACATAAAATTCCTGTGTAGGCTGGAG CTGCTT	Deletion of *spvD* in STm
spvDkop2	ATGAGAGTTTCTGGTAGTGCGTCATCCCAAGATATAATATCATATGAATATCCTC CTTAG	Deletion of *spvD* in STm
steAkop1	TTAATAATTGTCCAAATAGTTATGGTAGCGAGCTTTTATGTGTGTAGGCTGGAG CTGCTT	Deletion of *steA* in STm
steAkop2	ATGCCATATACATCAGTTTCTACCTATGCCAGAGCTTTATCATATGAATATCCTC CTTAG	Deletion of *steA* in STm
sseK3kop1	ATGTTTTCTCGAGTCAGAGGTTTTCTTTCATGCCAGAACTTGTGTAGGCTGGAG CTGCTT	Deletion of *sseK3* in STm
sseK3kop2	TTATCTCCAGGAGCTGATAGTCAAACTGCTGGTATCCATACATATGAATATCCTC CTTAG	Deletion of *sseK3* in STm
steEkop1	GTGATGAGATTCGTATATATTTATATCTTAGTGATTTATGTGTGTAGGCTGGAGC TGCTT	Deletion of *steE* in STm
steEkop2	TTATTCATCCGGGAAAACCTCTGCAGAATGCCTGTATTGACATATGAATATCCTC CTTAG	Deletion of *steE* in STm
TSP369	GTGATGAGATTCGTATATATTTATATCTTAGTGATTTATGGTGTAGGCTGGAGCT GCTTC	Deletion of *sarA* in STm
TSP370	ATCCGGGAAAACCTCTGCAGAATGCCTGTATTGAGCGATACATATGAATATCCT CCTTAG	Deletion of *sarA* in STm
srfJkop1	ATGAAAGGCAGACTCATCTCTTCCGATCCGTATCGTCAGGTGTAGGCTGGAGC TGCTTC	Deletion of *srfJ* in STm
srfJkop4	CTGGACATCGCGGTTATACACCACCAGCACACGCTCGCCTTCTTCCGGGGATC CGTCGA	Deletion of *srfJ* in STm
FEGP101	ATGCCAGCAGGAATTAAACCAATATTTATCAATAATATGATTAAGACCCACTTTC ACATT	Deletaion of *gogA* in STm
FEGP102	TCAATTACTAGATTCGTAGGCGATTCTTGGTGGTGATGTGCTAAGCACTTGTCT CCTG	Deletaion of *gogA* in STm
TSP540	CCATGTAATAAAAAGGATGTGTAACTCATCATGCCAACGGGTGTAGGCTGGAG CTGCTTC	Deletaion of *gtgA* in STm
FEGP106	TCAATTACTAAATTCGTAGGCGATTCTTGGTGGTGATGTGCATATGAATATCCTC CTTAG	Deletaion of *gtgA* in STm
TSP390	GGAAGGAACCAAGCTTTCGGCGCAGCTATTTATAACG	Construction of pTS11
TSP418	GGAAGGAACCGGTACCTGGTGAGGCTATTTCACACGAA	Construction of pTS11
JKP733	GTTCAGCAGCCACCTGCAATAATTCGTGTCGCTCAAG	Construction of pJK724

JKP734	GACCATGATTACGCCAATGTATTTAGAAAAATAAACAAAAAG	Construction of pJK724
JKP1033	ATTCGAGCTCGGTACCCGGGGTTTGACAGCTTATCATCGAC	Construction of pJK761, pJK762, pJK764, pJK766, pJK767, pJK768, pJK769 (pTrc 5')
JKP1034	AAAATATCATGGTCTGTTTCCTGTGTGAAATTG	Construction of pJK761 (pTrc 3')
JKP1035	GAAACAGACCATGATATTTTCGGTGCAGGAG	Construction of pJK761 (*avrA* 5')
JKP1036	CATGCCGGTCGACTCTAGAGTTAGCATAACGGCATTGTTATC	Construction of pJK761 (*avrA* 3')
JKP1039	GTGGGATCATGGTCTGTTTCCTGTGTGAAATTG	Construction of pJK766 (pTrc 3')
JKP1040	GAAACAGACCATGATCCCACCATTAAATAGATATGTTCCCG	Construction of pJK766 (*sseK1* 5')
JKP1041	CATGCCGGTCGACTCTAGAGCTACTGCACATGCCTCGCCC	Construction of pJK766 (*sseK1* 3')
JKP1044	AACGTGCCATGGTCTGTTTCCTGTGTGAAATTG	Construction of pJK767 (pTrc 3')
JKP1045	GAAACAGACCATGGCACGTTTTAATGCCGC	Construction of pJK767 (*sseK2* 5')
JKP1046	CATGCCGGTCGACTCTAGAGTTACCTCCAAGAACTGGCAG	Construction of pJK767 (*sseK2* 3')
JKP1049	GAGAAAACATGGTCTGTTTCCTGTGTGAAATTG	Construction of pJK768 (pTrc 3')
JKP1050	GAAACAGACCATGTTTTCTCGAGTCAGAGG	Construction of pJK768 (*sseK3* 5')
JKP1051	CATGCCGGTCGACTCTAGAGTTATCTCCAGGAGCTGATAG	Construction of pJK768 (*sseK3* 3')
JKP1054	CTGCTGGCATGGTCTGTTTCCTGTGTGAAATTG	Construction of pJK762 (pTrc 3')
JKP1055	GAAACAGACCATGCCAGCAGGAATTAAACC	Construction of pJK762 (*gogA* 5')
JKP1056	CATGCCGGTCGACTCTAGAGTCAATTACTAGATTCGTAGGC	Construction of pJK762 (*gogA* 3')
JKP1064	CCGTTGGCATGGTCTGTTTCCTGTGTGAAATTG	Construction of pJK764 (pTrc 3')
JKP1065	GAAACAGACCATGCCAACGGGAATTAAACC	Construction of pJK764 (*gtgA* 5')
JKP1066	CATGCCGGTCGACTCTAGAGTCAATTACTAAATTCGTAGGCG	Construction of pJK764 (*gtgA* 3')
JKP1076	AAACTCTCATGGTCTGTTTCCTGTGTGAAATTG	Construction of pJK769 (pTrc 3')
JKP1077	GAAACAGACCATGAGAGTTTCTGGTAGTGC	Construction of pJK769 (*spvD* 5')
JKP1078	CATGCCGGTCGACTCTAGAGTCAATCGTGTTTTTCATCATAAG	Construction of pJK769 (*spvD* 3')

JKP1084	ATTTCACACAGGAAACAGACCATGACATATAGATTGAAAAAGCG	Construction of pJK763 (*gogB* 5')
JKP1085	CGGGTACCGAGCTCGAATTCTCAACGATTTCTATTTTTAGGCTTATATTTATC	Construction of pJK763 (*gogB* 3')
JKP1086	ATTTCACACAGGAAACAGACCATGTTTAATATCCGCAATACAC	Construction of pJK765 (*sspH1* 5')
JKP1087	CGGGTACCGAGCTCGAATTCTCAGTTAAGACGCCACCG	Construction of pJK765 (*sspH1* 3')
5kbpIL10-1	TACGGACTAGTAATACGGTCAGACAGCGTCG	Construction of pWSK239+5kpbIL10
5kbpIL10-2	CTAATGGGCCCTGCAGTGGATCTGGACGAAG	Construction of pWSK239+5kpbIL10

REFERENCES

1. Singletary, L. A., Karlinsey, J. E., Libby, S. J., Mooney, J. P., Lokken, K. L., Tsolis, R. M., Byndloss, M. X., Hirao, L. A., Gaulke, C. A., Crawford, R. W., Dandekar, S., Kingsley, R. A., Msefula, C. L., Heyderman, R. S. & Fang, F. C. 2016. Loss of multicellular behavior in epidemic African nontyphoidal *Salmonella enterica* serovar Typhimurium ST313 Strain D23580. *mBio* **7**:e02265.

2. Ko, D.C., Shukla, K. P., Fong, C., Wasnick, M., Brittnacher, M. J., Wurfel, M. M., Holden, T. D., O'Keefe, G. E., Van Yserloo, B., Akey, J. M. & Miller, S. I. 2009. A genome-wide in vitro bacterial-infection screen reveals human variation in the host response associated with inflammatory disease. *Am. J. Hum. Genet.* **85**:214-217.

3. Jaslow SL, Gibbs KD, Fricke WF, Wang L, Pittman KJ, Mammel MK, Thaden JT, Fowler VG, Hammer GE, Elfenbein JR, Ko DC. 2018. *Salmonella* activation of STAT3 signaling by SarA effector promotes intracellular replication and production of IL-10. Cell Rep 23:3525–3536.

4. Karlinsey JE, Stepien TA, Mayho M, Singletary LA, Bingham-Ramos LK, Brehm MA, Greiner DL, Shultz LD, Gallagher LA, Bawn M, Kingsley RA. Libby SJ, Fang FC. 2019. Genome-wide analysis of *Salmonella enterica* serovar Typhi in humanized mice reveals key virulence features. Cell Host Microbe 26:426–434.

5. Alvarez, M.I., Glover, L. C., Luo, P., Wang, L., Theusch, E., Oehlers, S. H., Walton, E. M., Tram, T. T. B., Kuang, Y.-L., Rotter, J. I., McClean, C. M., Chinh, N. T., Medina, M. W., Tobin, D. M., Dunstan, S. J, & Ko, D. C. 2017. Human genetic

variation in *VAC14* regulates *Salmonella* invasion and typhoid fever through modulation of cholesterol. *Proc. Natl. Acad. Sci. U.S.A.* **114**:E7746-E7755.

6. Datsenko KA, Wanner BL. 2000. One-step inactivation of chromosomal genes in *Escherichia coli* K-12 using PCR products. Proc Natl Acad Sci U S A 97:6640–6645.

7. Berggren, R. E., Wunderlich, A., Ziegler, E., Schleicher, M., Duke, R. C., Looney, D. & Fang, F. C. 1995. HIV gp120-specific cell-mediated immune responses in mice after oral immunization with recombinant *Salmonella*. *J. Acquir. Immune Defic. Syndr. Hum. Retrovirol.* **10**:489–495.

8. Amann, E. & Brosius, J. 1985. 'ATG vectors' for regulated high-level expression of cloned genes in *Escherichia coli*. *Gene* **40**:183–190.

9. Pujol, C. & Bliska, J.B. 2003. The ability to replicate in macrophages is conserved between *Yersinia pestis* and *Yersinia pseudotuberculosis*. *Infect. Immun.* **71**:5892-5899.

10. Wang, R.F. & Kushner, S.R. 1991. Construction of versatile low-copy-number vectors for cloning, sequencing and gene expression in *Escherichia coli*. *Gene* **100**:195-199.
